# Acupressure and hypnosis in arterial hypertension: a series of N-of-1 trials

**DOI:** 10.3389/fmed.2026.1871793

**Published:** 2026-08-17

**Authors:** Miriam Ortiz, Max Marco Schreiner, Nicolas Volz, Tatjana Tissen-Diabaté, Eva Jansen, Julia Siewert, Michael Teut, Benno Brinkhaus

**Affiliations:** 1 Institute of Social Medicine, Epidemiology and Health Economics, Charité Universitätsmedizin Berlin, Freie Universität Berlin and Humboldt-Universität zu Berlin, Berlin, Germany; 2 Institute for Social Medicine and Epidemiology, Medizinische Hochschule Brandenburg Theodor Fontane, Brandenburg an der Havel, Germany

**Keywords:** acupressure, arterial hypertension, complementary medicine, hypnosis, integrative medicine, N-of-1 trial

## Abstract

**Background:**

Acupressure and hypnosis are used by patients with arterial hypertension despite limited evidence supporting their efficacy. The aim of this N-of-1 series was to evaluate feasibility, individual patient responses to both interventions, to inform personalized treatment decisions, and to assess overall effects of interventions.

**Methods:**

In a series of N-of-1 trials, we compared self-administered acupressure plus routine care (RC), hypnosis plus RC, and RC alone in patients with grade 1 or 2 hypertension. After a one-week run-in period with RC only, patients entered a six-week intervention phase consisting of two blocks with periods of acupressure, hypnosis and RC only, each followed by washout periods, administered in randomized order. Outcomes included systolic and diastolic blood pressure and pulse rate, which were measured by patients according to a standardized protocol. Patients recorded cardiovascular parameters, stress levels, vitality, and well-being using visual analogue scales, and documented feasibility and safety data in a study diary. The primary analysis of continuous outcomes was descriptive and conducted at the individual trial level. Secondary analyses included meta-analysis across all N-of-1 trials and outcomes. Overall group effects were analyzed using mixed-effects ANCOVA models.

**Results:**

Altogether 17 patients (59% female, mean age 54.4 [SD 7.6]) were included in this study. Feasibility of interventions was high, as reflected by an adherence rate exceeding 90%. Two patients showed reductions in systolic and diastolic blood pressure with both acupressure and hypnosis, and one patient with acupressure only, compared to RC. In the meta-analysis, no overall effects of acupressure or hypnosis on cardiovascular outcomes were observed. Overall, stress levels were higher during the run-in period (mean 43.6 [SD 18.9]) compared to the subsequent blocks and were similar between treatment periods. All other outcomes remained unchanged. Reported side effects were mild, rare, and occurred mainly during acupressure.

**Conclusion:**

This series of N-of-1 RCTs was feasible for self-administered acupressure and hypnosis for hypertension. No relevant changes in outcome parameters, including blood pressure, were observed at the group level, although selected patients showed individual changes. Further studies are needed to identify the treatment as well as individual conditions under which clinical benefits can be expected.

**Clinical trial registration:**

Deutsches Register für klinische Studien (DRKS) 00034937, https://drks.de/search/de/trial/DRKS00034937/details.

## 1 Background

Hypertension is one of the most common diseases worldwide. A comprehensive global analysis of 1,201 studies found that the global age-standardized prevalence hypertension is 32% in women and 34% in men aged 30–79 years (1). Besides established pharmacological treatments, non-pharmacological interventions play an important role in the treatment and prevention of hypertension. Two recently published hypertensions guidelines emphasize the importance of these approaches, strongly recommending lifestyle modifications that include improved stress management, for example through relaxation techniques (2, 3). Several interventions from Traditional Complementary and Integrative Medicine (TCIM) are key elements of non-pharmacological hypertension management, including relaxing mind–body therapies, yoga, and acupuncture, although evidence from RCTs often remain inconclusive (4).

Acupressure is a technique from Chinese Medicine and relates to the manual stimulation of acupuncture points. In contrast to acupuncture, it can be self-administered, it is easy to learn and a safe practice for patients in several conditions (5–8). Several small and heterogeneous studies have reported effects of self-administered acupressure on blood pressure (BP), compared with sham acupressure or moxibustion, either after a single intervention (8, 9) or following repeated acupressure over eight weeks (10).

For acupuncture, which is considered to be more effective than acupressure, effects on hypertension are also uncertain. A Cochrane meta-analysis found significant short-term effects on BP but stated that there was no overall evidence of a sustained BP-lowering effect of acupuncture that would be beneficial for the management of chronically elevated BP (11). For grade 1 hypertension, another review also suggested potential effects of acupuncture; however, the substantial heterogeneity of the included studies limits the generalizability of these findings (12, 13).

Hypnosis is an established method used in many areas of medicine including psychotherapy, to, for example, treat anxiety, depression, and stress, as well as to provide pain relief, help with smoking cessation, and support patients during childbirth, and dental procedures (14–18). Therapeutic suggestions are used to induce a trance in patients. Once this trance state is achieved, the therapist can use further suggestions to bring about changes or alleviate symptoms, while the patient’s mental imagery is guided by the therapist’s suggestions. An RCT (*n* = 64) comparing 15 min of self-administered hypnosis by listening to audio recordings versus resting showed good feasibility and a significant reduction in systolic BP and stress levels in patients with hypertension (19). In addition, mid- and long-term (effects up to one year) have been found for hypnosis combined with self-administered hypnosis compared to a control group in an RCT with 31 participants (20).

However, despite its widespread use and recommendation in clinical guidelines, there is only limited evidence of sustained, clinically significant effects of self-administered acupressure and hypnosis in patients with chronic hypertension. Previous studies have focused primarily on collective effects under strictly controlled conditions. There have been few reports to date on interindividual differences in responses to these interventions. Furthermore, little is known about the feasibility of self-administered hypnosis and acupressure under real-world conditions.

The aims of this pilot study were to (a) evaluate the feasibility of conducting a series of N-of-1 trials in patients with arterial hypertension; (b) compare the individual-level effects of acupressure or hypnosis on systolic and diastolic BP, pulse rate, perceived stress, vitality and well-being, and (c) assess the aggregated treatment effects across the whole study cohort.

## 2 Materials and methods

### 2.1 Design

This pilot study comprised a series of randomized N-of-1 trials comparing the feasibility and potential effects of acupressure plus routine care (RC), hypnosis plus RC, and RC alone in patients with hypertension.

**Figure 1:**
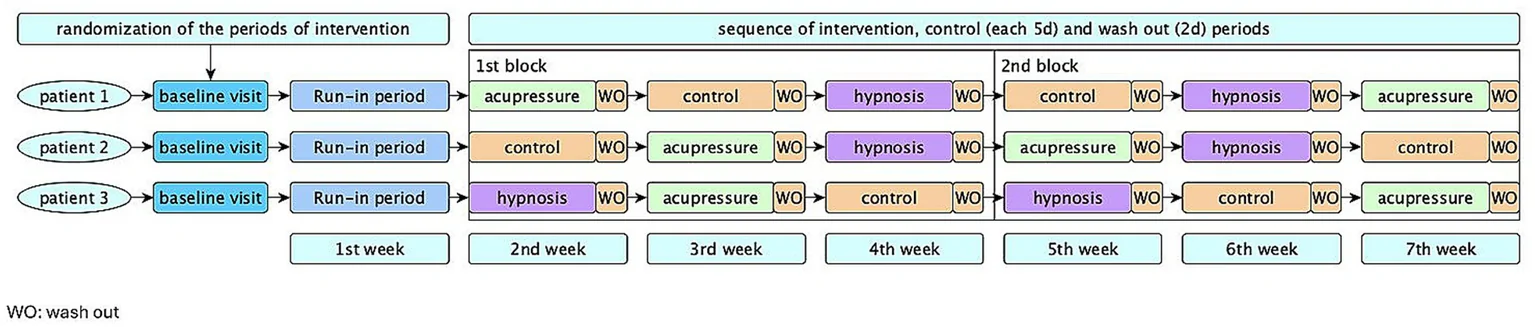
Study design.

The study duration for each patient was seven weeks and started with a 7-day run-in period with RC only, intended to strengthen measurement and documentation routines and to gather baseline values. This was followed by two blocks of randomized treatment periods, each lasting five days, comprising acupressure plus RC, hypnosis plus RC, and RC alone. Each intervention period was followed by a washout period of 2 days (see Figure 1). Since we assumed, based on experience, rather short-term effects of acupressure and hypnosis, this period seemed to be sufficient to reduce or eliminate the effects of the previous intervention. The study included a qualitative part with two focus groups after the completion of all N-of-1 trials. Mixed methods study results will be reported elsewhere.

Randomization for the N-of-1 studies with two active interventions and one control condition, was performed in a 1:1:1 allocation ratio with two blocks. To generate the randomization schedule, we used R (version 4.4.1; R Core Team, 2025; R Foundation for Statistical Computing, Vienna, Austria) with a pre-specified block randomization algorithm. Each patient was assigned one of six possible sequences within each block to ensure even distribution. Allocation was concealed. The randomization order was communicated by phone from the study center to the study physician after the patients had been informed, provided written consent, had been examined for eligibility, and had completed the baseline questionnaire. The result of the randomization was then communicated to the patients. Each patient was assigned a patient number and given a study diary, which was structured in accordance with the randomized sequence of intervention and control phases.

The trial was conducted at the Charité outpatient clinic for Complementary and Integrative Medicine in Berlin between September 2024 and February 2025. A vote of the responsible ethics committee (Ethics Committee of the Charité - Universitätsmedizin Berlin) was obtained before the study start (EA1/108/24, 2024-08-06) and the study has been registered in the German registry of clinical trials (DRKS00034937). All study patients were informed individually about the study and provided written informed consent before study inclusion. Patients received a EUR 20 shopping voucher as an incentive and could keep the automatic BP monitor, provided for the measurements. After completion of study, each patient was sent an individualized report summarizing their results, as well as the aggregated results for the whole sample, presented in both text and graphical formats (Supplementary material: Information for patients’ results). A phone call with the study physician was offered to discuss the meaning of the results.

### 2.2 Patients

Patients were recruited mainly by digital media and posters at the University Campus of the Charité - Universitätsmedizin Berlin. The inclusion criteria were as follows: persons of any sex and gender, age 30 to 65 years, a diagnosis of primary hypertension stage 1 or 2, upper arm circumference between 22 and 32 cm. The main exclusion criteria were the use of more than two classes of antihypertensive medication, known secondary arterial hypertension, further severe cardiovascular diseases (such as coronary heart disease) and arrhythmia, known mental and chronic pain disease, pregnancy, use of acupressure, acupuncture, hypnosis, or relaxation techniques during the last 3 months prior to the study.

### 2.3 Study interventions

Since the aim of the study included investigating the feasibility of self-administered home-based interventions that were not delivered and controlled by the study staff during the study, patients were prepared accordingly. Patients were trained during the baseline visit in acupressure and informed about both interventions. A link sent at the end of the run–in period gave patients access to written educational information about acupressure and hypnosis, films for conducting acupressure and hypnosis, and to the audio file with which the hypnosis intervention was to be carried out. Each patient received a structured study diary corresponding to the randomized intervention or control periods, which clearly indicated the current treatment period and was supplemented by email reminders from the study center. Patients were encouraged to contact the study center in case of any query.

#### 2.3.1 Acupressure

**Figure 2:**
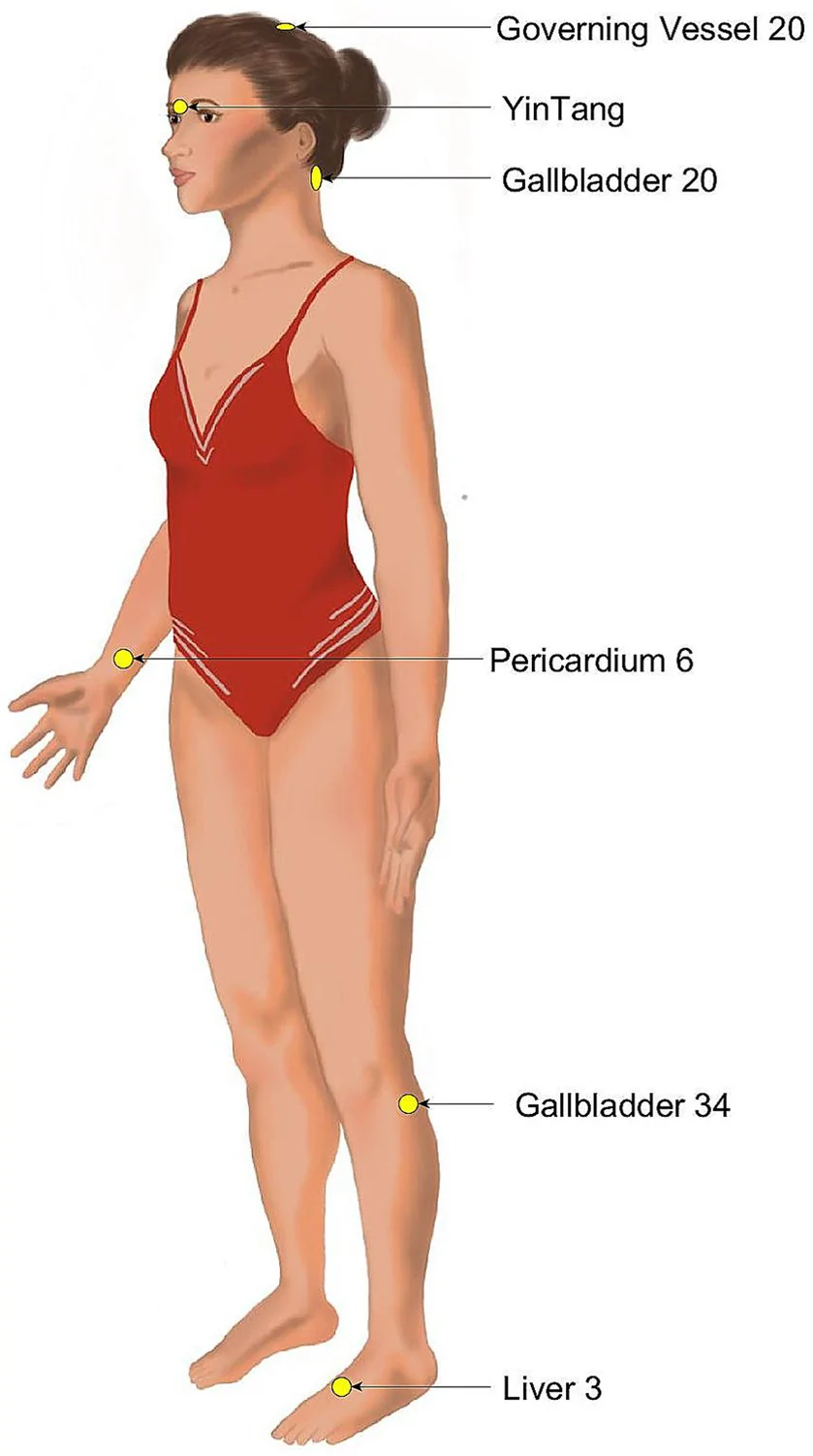
Acupuncture point location.

Four experienced Chinese Medicine (CM) experts from the Charité outpatient clinic for complementary and Integrative Medicine developed the standardized acupressure procedure for this trial. This included the choice of six acupressure points (bilateral: Liver 3, Gallbladder 34 and 20, Pericardium 6, Gouvernor Vessel 20, Yin Tang) as well as the duration, frequency and intensity of acupressure (Figure 2). Each patient received thorough individual acupressure training of 20 min duration, including an introduction to CM and a demonstration of the acupressure points and technique as well as detailed instructions in written form and a video for domestic use, given by a study physician qualified in CM. During the acupressure periods, patients were required to apply acupressure daily at all predefined points for approximately 20 min per day. This could be performed either as two 10-min sessions or as one 20-min session.

#### 2.3.2 Hypnosis

The self-administered hypnosis consisted of listening to a hypnotherapeutic audio file which was based on an established deep trance hypnosis script “Three-level-trance” (21). The audio induced relaxation and trance in a stepwise manner by guiding breathing and progressively slowing the breath, followed by deepening relaxation across three stages. Suggestions focused on relaxation and well-being. Each session concluded with a posthypnotic suggestion and reorientation. Patients were encouraged to self-administer hypnosis once daily for 20 min by using the audio file.

All patients were instructed to continue their RC including a maximum of two different anti-hypertensive medications. During control periods, patients received RC only.

### 2.4 Feasibility and outcome measurements

Feasibility was considered in relation to the patients` recruitment, treatment - specific adherence and retention, adherence to the randomized sequence and treatment-specific fidelity rates (22, 23).

Cardiovascular outcomes comprised systolic and diastolic BP (mmHg) and pulse rate. BP and pulse rate were measured by patients twice daily, in the morning and evening, and recorded in a paper-based study diary so that compliance with the given measurement frequency could be assessed. The measurement was carried out once per timepoint (morning/evening, daily) using a standardized automatic oscillometric blood pressure monitor (Boso Medicus X) in mmHg. This device has a measurement accuracy according to ISO 81060-2, the maximum measurement deviation of the cuff pressure is ± 3 mmHg or 3% of the reading value (whichever is the greater value). The patients received identical devices (Boso Medicus X) and precise oral and written instructions for the measurement from the study staff during the baseline visit. The first BP measurement was performed by the study staff and used to demonstrate the procedure exactly. Before the measurement, patients should sit for 5 min in a relaxed posture (back and arms leaning, legs not crossed, feet flat on the floor) in a quiet environment. The cuff should be tightly applied to the undressed upper arm at heart level for measurement. The bottom edge of the cuff should be 2–3 cm above the crook of the elbow, and the mark had to be on the artery. BP was measured throughout the study on the arm where it was highest at the baseline visit. During the measurements, the patient was not allowed to move (not even speak) and the arm was to be placed slightly bent. Further, abstinence from caffeine and nicotine one hour before BP measurement was recommended as well as a measuring always at the same time daily in the morning and evening. The timing between the measurement and the intervention was not specified.

Additional exploratory outcomes included subjectively perceived stress levels, vitality and well-being, each measured once daily in the evening by the patient using a visual analogue scale (VAS, 0–100 mm). The VAS is an established and validated method for self-assessment (24, 25), e.g., for measuring pain or general discomfort (26). Patients marked their current stress level on a 100 mm line ranging from 0 (‘no stress’) to 100 (‘maximum conceivable stress’). Like stress, vitality and well-being were measured by a VAS. Vitality is equated with ‘life vigor’, referring to the ability of an organism to respond adequately to changing environments, encompassing both physical and mental energy. This definition was also communicated to the patients. Wood et al. measured physical and mental vitality on a VAS (27). A VAS for subjective well-being was first used by Gall et al. (28). For both vitality and well-being, higher values indicate a better status.

All continuous outcome values were calculated as the mean of measurements over each 5-day assessment period.

Therapy-related adverse events and the feasibility, intensity and frequency of acupressure and hypnosis performance were assessed with patients’ diaries during the two blocks. In addition, standardized questions assessing the expected effectiveness of acupressure and hypnosis were included in the baseline questionnaire.

### 2.5 Statistics

#### 2.5.1 Sample size

This pilot study treated each patient as an individual N-of-1 randomized controlled trial. No formal sample size calculation was performed. It was estimated that 10 patients would be sufficient to make a statement about the feasibility of the interventions and the study. Given the anticipated burden of daily interventions, blood pressure measurements, and documentation (approximately 30 min per day), reduced adherence and dropouts were expected. Based on an assumed dropout rate of at least 30% (29), a target sample size was set at a minimum of 13 patients.

#### 2.5.2 Data analysis

The quantitative data were analyzed using SPSS (IBM SPSS Statistics, Version 29) and R (R Core Team, 2024), Version 4.4.2 or higher. All collected data were analyzed descriptively. Continuous variables are presented as means ± standard deviation or as medians with interquartile ranges, and categorical variables as frequencies and percentages. The results are reported separately for the treatments, for blocks and overall. Time series are presented graphically. For outcomes measured twice daily, daily mean values were calculated.

The primary analysis of continuous endpoints was descriptive for each individual study. To summarize data across studies and compare interventions, mixed-effects ANCOVA models were applied, with treatment (acupressure, hypnosis, control) included as a factor and the respective baseline value (mean of the run-in period) as a covariate. Individual patients were included as random effects. Adjusted mean values and mean differences, each with a 95% confidence interval (CI) were reported. Model diagnostics were assessed graphically. The normality assumption of the model residuals was assessed using Q-Q plots. In addition, residuals-versus-fitted plots were inspected to evaluate potential non-linearity and heteroscedasticity. No substantial deviations from the assessed model assumptions were identified.

For additional secondary analyses, a meta-analysis of all N-of-1 studies with available data (mean differences, variability) for the respective outcomes was performed. Carryover effects were inspected graphically and descriptively, using changes in outcomes between last measurement of intervention period and last measurement of washout period as well as in comparison to baseline.

Subgroup analyses were pre-specified for hypertension grade, sex, age (<50), and antihypertensive medication. All *p*-values were considered exploratory without adjustment for multiple testing. The evaluation was performed according to the intention-to-treat principle, without replacing missing values.

Side effects, adverse events, number of interventions performed, and changes/deviations from RC were evaluated descriptively. Prior to data analysis, a statistical analysis plan was prepared, providing detailed specifications of the planned analyses.

## 3 Results

**Figure 3:**
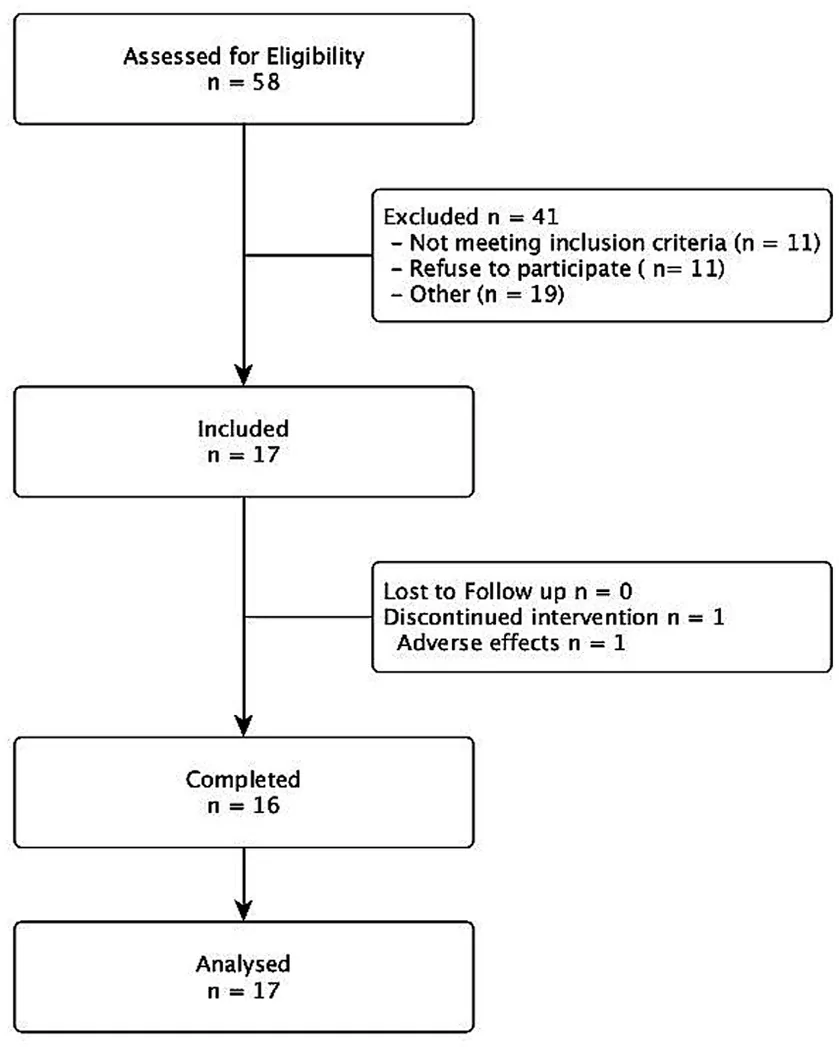
Patients flow chart.

We included 17 patients between September and December 2024 in this study. One patient dropped out due to a serious adverse event which was not related to one of the study interventions and led to an interruption of the self-administered intervention and measurements in the second block of her N-of-1-trial (see Figure 3).

### 3.1 Baseline characteristics

Baseline characteristics are shown in Table 1. The sample (59% female) consisted of middle aged (range 36–63), working, physically active, and slightly overweight patients who were mostly higher educated. Mean duration of hypertension history was 8 years (median (IQR): 5 (3, 10)). Cardiovascular parameters at baseline were within normal range for BP and pulse rate. Most patients suffered from grade I hypertension (*n* = 15, 88.2%), based on BP measurements obtained at the study center at baseline and values reported in their medical history. Two patients suffered from grade 2 hypertension and took no antihypertensive medication. From the remaining patients, *n* = 5 took angiotensin-converting enzyme inhibitors, *n* = 8 angiotensin-converting enzyme subtype 1 inhibitors, *n* = 4 calcium channel blockers (dihydropyridines), *n* = 2 diuretics and *n* = 2 betablocker; *n* = 8 had a combined medication (Supplementary Table 1). Mean VAS scores indicated moderate levels of perceived stress (mean (SD) 43.6 (18.9)), vitality (57.8 (14.8)), and well-being (63.0 (16.5)).

**Table 1:** Baseline characteristics and cardiovascular measurements from the run-in period.

Characteristics	Total *N* = 17
	Mean (SD)/*n* (%)
Sex	
Female	10 (58.8%)
Male	7 (41.2%)
Age	54.4 (7.6)
BMI	28.0 (5.3)
Overweight or adipositas (BMI ≥ 25)	12 (70.6%)
Years with hypertension	8.2 (7.5)
Hypertension Grade I	15 (88.2%)
Hypertension Grade II	2 (11.8%)
Lifestyle habits	
Smokers	1 (5.9%)
Physical activity: MET-minutes (intensive work, transport and leasure time)	3,470.6 (3,380.5)
Physical active: WHO-criterium of 600 MET-minutes/week fulfilled	15 (88.2%)
Education	
Secondary school diploma/elementary school diploma	1 (5.9%)
Polytechnic secondary school/10th grade	4 (23.5%)
High school degree	11 (64.7%)
Other	1 (5.9%)
Highest professional qualification	
other (eg. vocational training)	3 (17.6%)
Vocational school, technical college	4 (23.5%)
College education, university	10 (58.8%)
Currently employed	17 (100%)
Positive expectancy for hypnosis on lowering BP	17 (100%)
Positive expectancy for acupressure on lowering BP	15 (88.2%)
Run-in period (7 days)*	
Morning systolic BP (mmHg)	131.9 (13.6)
Morning diastolic BP (mmHg)	86.8 (7.4)
Evening systolic BP (mmHg)	134.5 (12.5)
Evening diastolic BP (mmHg)	86.6 (6.4)
Morning pulse rate (bpm)	70.1 (9.3)
Evening pulse rate (bpm)	72.5 (9.2)
Perceived Stress (VAS, 1–100 mm)	43.6 (18.9)
Vitality (VAS, 1–100 mm)	57.8 (14.8)
Well-being (VAS, 1–100 mm)	63.0 (16.5)

*mean of measurements over 7 days.

SD, standard deviation; BP, blood pressure; VAS, visual analogue scale; bpm, beats per minute; BMI, body mass index; MET, metabolic equivalent of task.

### 3.2 Feasibility and adherence

The overall adherence to the study procedures was very good. Across the four intervention periods (two in each block), the intervention was performed on 98% of the allocated days (320/328, missing data for 12 days). Almost all of the missing data were observed for the patient that dropped out. Only 5 days (3%) without acupressure and 3 days (1,9%) without hypnosis during the, respectively, intervention periods have been observed. This reflects an adherence of more than 93%. During the first block, three patients performed the acupressure twice daily on a single occasion; on all other days, they performed it once daily, consistent with the other patients. 88% performed acupressure and 94% hypnosis in the evening. Antihypertensive medication remained stable during the study.

For the measuring of BP and pulse rate, morning measurements were completed by 97% of the patients and evening measures by 95%. Patients could decide if they measured before or after study intervention. Mean morning and evening measuring times were 7.40 a.m. and 9.00 p.m. Measurement times were generally consistent during the first study weeks but showed greater within-participant variation in the later weeks (Supplementary Figure 6). Mainly, patients measured cardiovascular parameters after the intervention (82% after acupressure and 57% after hypnosis).

### 3.3 Individual treatment effects (N-of-1 level)

A reduction in systolic and diastolic BP was observed in three patients, either in one or both intervention periods. Sociodemographic data of these two males and one female patient were very heterogeneous in terms of age, BMI, salt consumption, education, treatment expectations before the study; their baseline and run in blood pressure and pulse rate were comparable. All of them were under hypertensive medication (Supplementary Table 2).

### 3.4 Meta-analyses of N-of-1-trials

**Figure 4:**
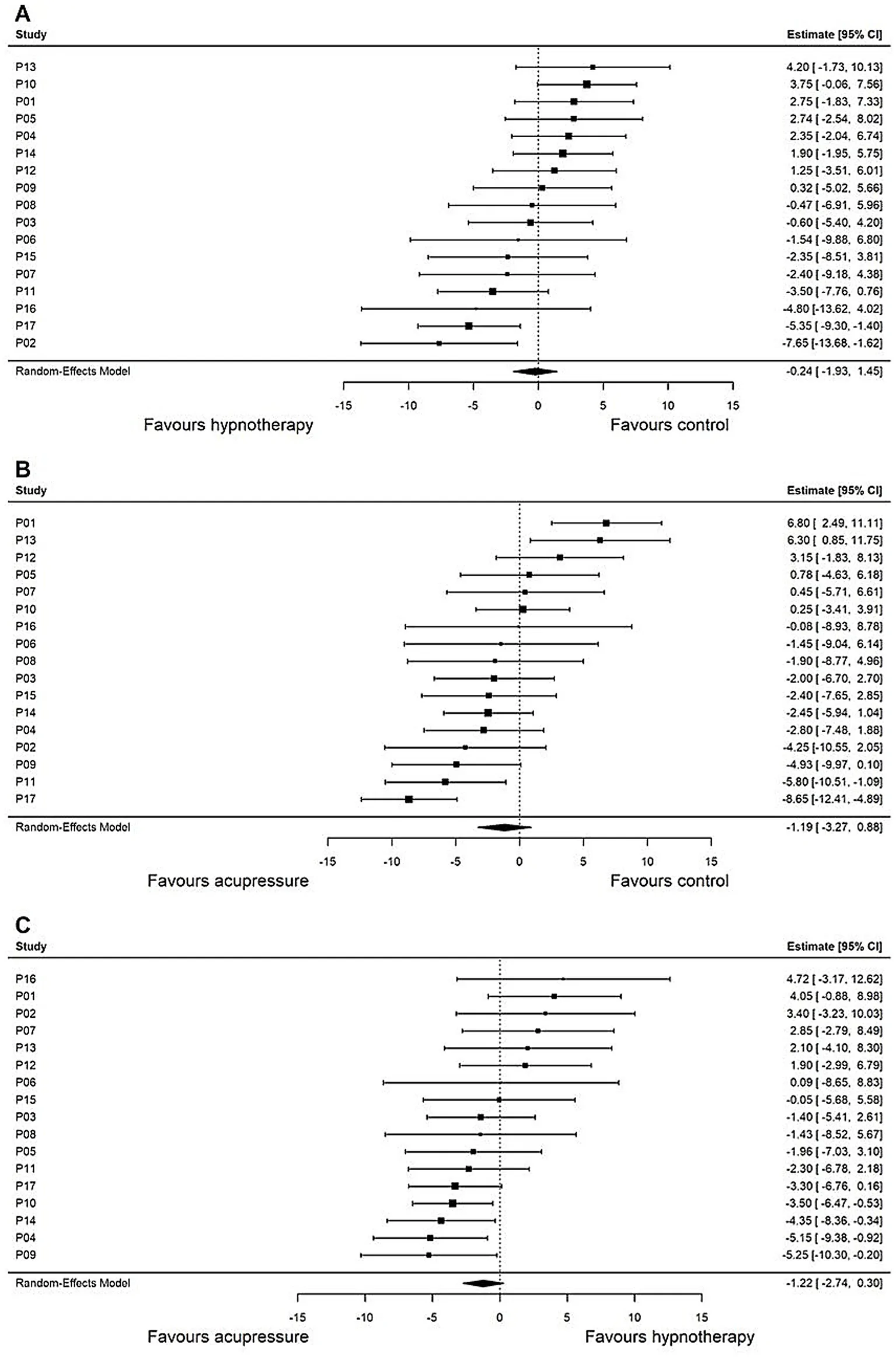
Forest plots from random-effects meta-analyses of systolic blood pressure (mean differences) across treatment comparisons **(A–C)**.

**Figure 5:**
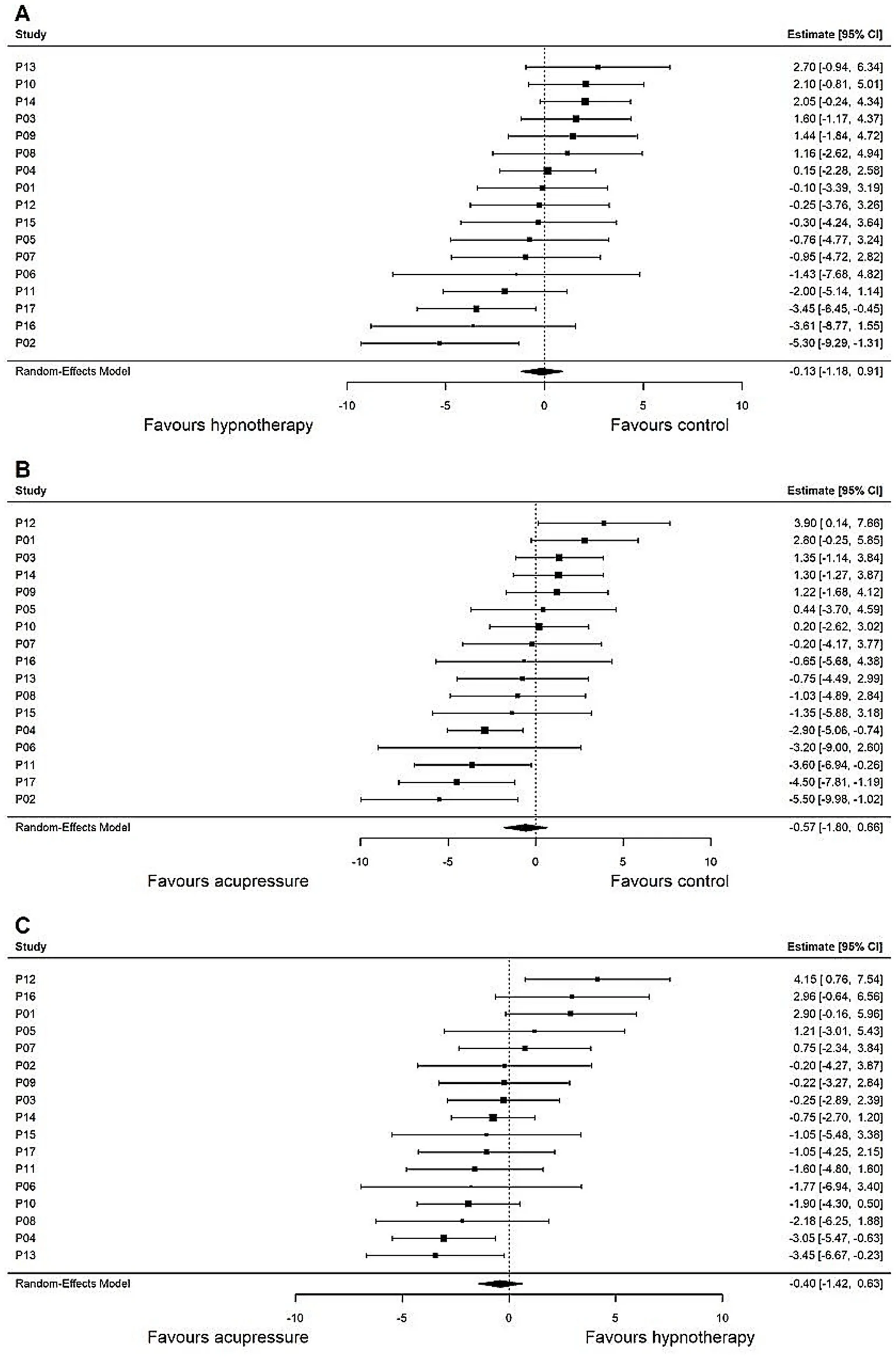
Forest plots from random-effects meta-analyses of diastolic blood pressure (mean differences) across treatment comparisons **(A–C)**.

The meta-analysis of the N-of-1 trials showed substantial heterogeneity in treatment effects. Comparisons of hypnosis versus RC and acupressure versus RC revealed a wide range of effects, spanning from favoring the intervention to favoring the control condition. Inspection of the forest plots indicated that some individual trials showed results in favor of one of the interventions compared to control, primarily for BP outcomes. For systolic BP, two patients each showed lower values during acupressure (patients #17 and #11) and hypnosis (patients #17 and #02) compared to control periods (Figure 4). For diastolic BP, four patients (#04, #11, #17, and #02) showed lower values during acupressure, and two patients (#17 and #02) during hypnosis, compared to control. Interestingly, patients #02 and #17 appeared to respond to both acupressure and hypnosis with reductions in systolic and diastolic BP, whereas patient #11 showed a response only to acupressure (Figure 5).

The meta-analysis did not show any consistent differences between acupressure and control, hypnosis and control, or acupressure and hypnosis at the group level. This finding was consistent across all outcomes (Figures 4, 5; Supplementary Figures 1–4).

### 3.5 Across-patient effects (mixed-effects models)

**Figure 6:**
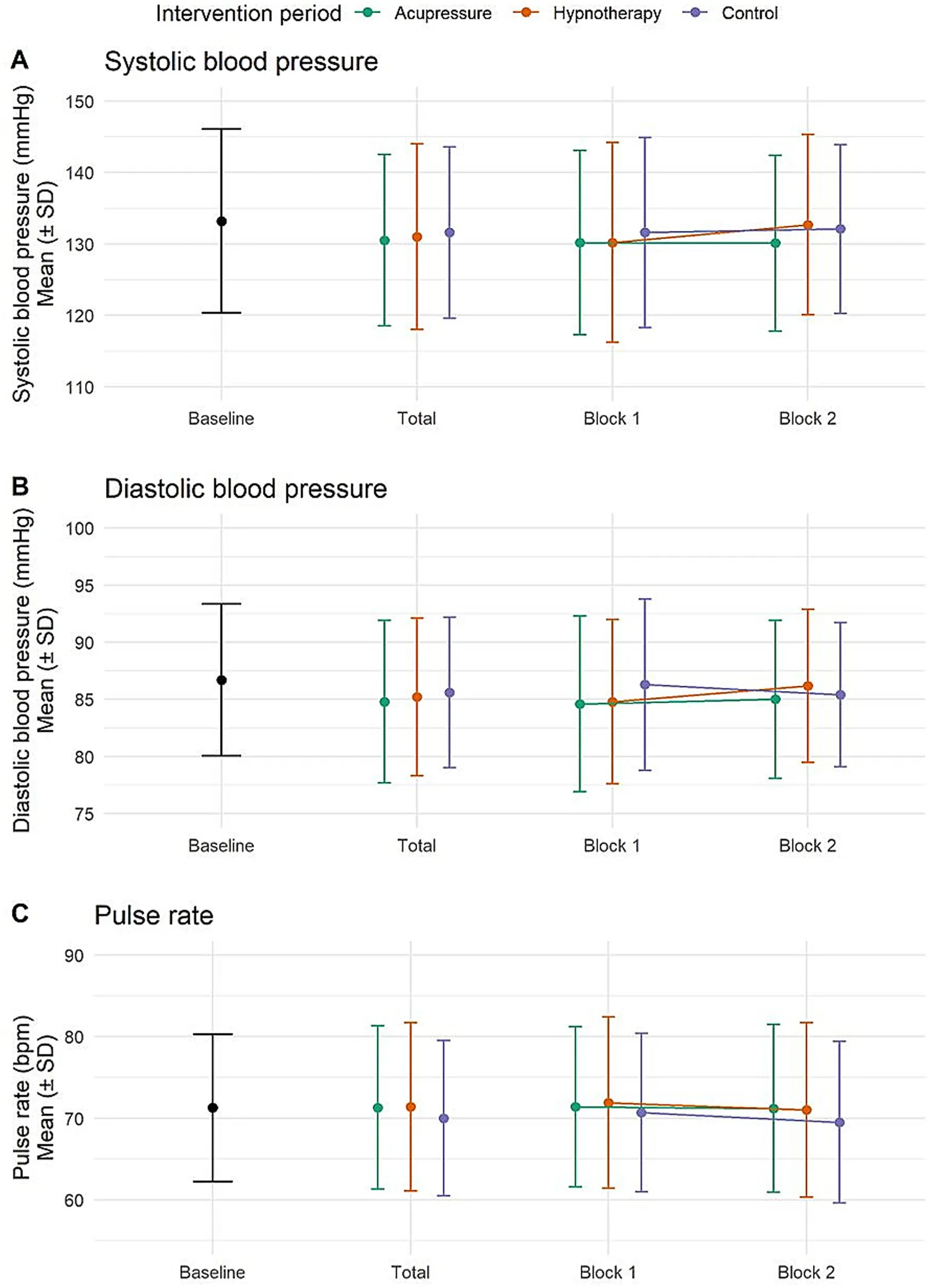
Baseline and follow-up mean values (± one standard deviation) for BP (daily means derived from morning and evening measurements) and pulse rate **(A–C)**.

Cardiovascular outcomes for the whole sample showed only minor and clinically non-relevant differences between study intervention periods and the RC control periods over the course of the study. This was consistent across both the descriptive analyses (Table 2) and the adjusted linear mixed-effects models (Table 3; Figure 4). Means of BP and pulse rates were in the high to normal ranges of BP, indicating a possibly overall effective medication. During the intervention periods, systolic BP was slightly higher in the evening than in the morning (Tables 2, 3). Mean systolic and diastolic BP values were slightly lower during the acupressure periods, but variability was considerable (e.g., diastolic BP SD range 8.1–9.4 mmHg) (Figure 6).

**Table 2:** Descriptive analysis of BP, pulse rate and subjective rating of stress, vitality and well-being.

Outcome	Daytime	Akupressure periods			Hypnosis periods			Control periods		
		1. Block	2. Block	Mean	1. Block	2. Block	Mean	1. Block	2. Block	Mean
		Mean (SD)	Mean (SD)	Mean (SD)	Mean (SD)	Mean (SD)	Mean (SD)	Mean (SD)	Mean (SD)	Mean (SD)
Systolic BP (mmHg)	Morning	128.7 (14.6)	128.5 (13.3)	128.6 (13.9)	129.0 (15.8)	130.8 (14.6)	129.9 (15.2)	131.2 (16.0)	131.1 (13.8)	131.1 (14.9)
	Evening	132.0 (14.8)	133.0 (13.5)	132.5 (14.2)	131.6 (15.7)	134.7 (14.7)	133.1 (15.2)	131.6 (14.1)	132.8 (13.4)	132.2 (13.7)
	Mean	130.3 (14.8)	130.7 (13.5)	130.5 (14.2)	130.3 (15.7)	132.7 (14.7)	131.5 (15.3)	131.4 (15.1)	131.9 (13.6)	131.7 (14.3)
Diastolic BP (mmHg)	Morning	84.8 (8.8)	85.0 (8.5)	84.9 (8.7)	85.1 (8.4)	85.7 (8.7)	85.4 (8.6)	86.6 (8.7)	85.8 (8.5)	86.2 (8.6)
	Evening	84.3 (9.4)	85.7 (8.6)	85.0 (9.0)	84.5 (8.3)	86.6 (8.3)	85.5 (8.3)	86.0 (10.1)	84.8 (7.6)	85.4 (9.0)
	Mean	84.6 (9.1)	85.4 (8.6)	85.0 (8.8)	84.8 (8.3)	86.2 (8.5)	85.5 (8.4)	86.3 (9.4)	85.3 (8.1)	85.8 (8.8)
Pulse rate	Morning	69.6 (11.7)	69.8 (11.7)	69.7 (11.7)	70.5 (11.1)	69.8 (12.0)	70.2 (11.5)	69.5 (11.1)	68.3 (10.6)	68.9 (10.8)
	Evening	73.3 (11.0)	72.5 (12.1)	72.9 (11.5)	73.2 (12.5)	71.9 (11.4)	72.5 (12.0)	71.9 (10.8)	70.9 (11.5)	71.4 (11.1)
	Mean	71.5 (11.5)	71.2 (12.0)	71.3 (11.7)	71.8 (11.9)	70.8 (11.7)	71.3 (11.8)	70.7 (11.0)	69.6 (11.1)	70.1 (11.0)
Perceived Stress (VAS 1–100 mm)	—	37.5 (18.5)	33.1 (23.8)	35.4 (21.3)	41.1 (22.5)	30.0 (21.9)	35.7 (22.8)	40.0 (20.0)	29.2 (21.0)	34.8 (21.1)
Vitality (VAS 1–100 mm)	—	62.3 (15.2)	60.3 (19.3)	61.3 (17.3)	57.6 (20.8)	58.7 (22.2)	58.1 (21.4)	56.0 (17.1)	58.8 (19.6)	57.4 (18.4)
Well-being (VAS 1–100 mm)	—	63.0 (16.7)	62.4 (18.9)	62.8 (17.7)	61.4 (21.1)	61.7 (23.2)	61.5 (22.1)	58.5 (18.8)	62.6 (18.8)	60.5 (18.9)

BP, blood pressure; SD, standard deviation.

**Table 3:** Blood pressure, pulse rate frequency and subjective rating of stress, vitality and well-being.

Outcomes	Daytime	Acupressure periods			Hypnosis periods			Control periods		
		1. Block	2. Block	Overall	1. Block	2. Block	Overall	1. Block	2. Block	Overall
		aMean (95% CI)	aMean (95% CI)	aMean (95% CI)	aMean (95% CI)	aMean (95% CI)	aMean (95% CI)	aMean (95% CI)	aMean (95% CI)	aMean (95% CI)
Systolic BP (mmHg)	Morning	128.5 (125.8–131.2)	128.4 (125.3–131.4)	128.4 (126.3–130.6)	129.0 (126.3–131.7)	130.2 (127.1–133.3)	129.5 (127.3–131.6)	131.2 (128.5–133.9)	130.7 (127.5–133.8)	131.0 (128.8–133.1)
	Evening	132.1 (128.9–135.2)	133.0 (129.8–136.2)	132.6 (129.8–135.3)	131.5 (128.3–134.6)	134.3 (131.1–137.6)	132.6 (129.8–135.4)	131.9 (128.8–135.1)	132.6 (129.4–135.8)	132.1 (129.4–134.9)
	Mean	130.3 (127.7–132.8)	130.7 (128.0–133.4)	130.5 (128.4–132.5)	130.2 (127.7–132.8)	132.2 (129.5–135.0)	131.0 (129.0–133.1)	131.6 (129.0–134.1)	131.6 (128.9–134.3)	131.5 (129.5–133.6)
Diastolic BP (mmHg)	Morning	84.8 (82.9–86.7)	85.0 (82.5–87.4)	84.9 (82.9–86.8)	85.1 (83.2–87.0)	85.3 (82.8–87.8)	85.1 (83.2–87.0)	86.6 (84.7–88.5)	85.5 (83.1–88.0)	86.1 (84.1–88.0)
	Evening	84.4 (82.3–86.4)	85.9 (83.8–87.9)	84.8 (83.1–86.6)	84.5 (82.5–86.6)	86.8 (84.7–88.9)	85.2 (83.5–87.0)	85.9 (83.8–88.0)	84.9 (82.8–87.0)	85.0 (83.3–86.7)
	Mean	84.6 (83.0–86.2)	85.4 (83.4–87.4)	84.8 (83.3–86.4)	84.8 (83.2–86.5)	86.1 (84.1–88.1)	85.2 (83.6–86.7)	86.2 (84.6–87.9)	85.3 (83.3–87.2)	85.5 (84.0–87.1)
Pulse rate	Morning	69.5 (67.4–71.7)	70.0 (67.9–72.2)	69.6 (67.7–71.5)	70.5 (68.3–72.6)	70.0 (67.8–72.2)	70.1 (68.2–72.0)	69.5 (67.4–71.7)	68.4 (66.2–70.6)	68.8 (66.9–70.7)
	Evening	73.3 (70.9–75.6)	72.7 (70.8–74.6)	72.9 (71.0–74.8)	73.3 (71.0–75.7)	72.2 (70.3–74.1)	72.6 (70.7–74.5)	71.9 (69.6–74.3)	70.8 (68.8–72.7)	71.4 (69.5–73.3)
	Mean	71.4 (69.5–73.3)	71.4 (69.6–73.1)	71.2 (69.6–72.9)	71.9 (70.0–73.8)	71.1 (69.4–72.9)	71.4 (69.7–73.0)	70.7 (68.8–72.7)	69.6 (67.8–71.4)	70.1 (68.4–71.7)
Perceived Stress (VAS)	—	37.5 (31.6–43.3)	35.2 (27.5–42.9)	35.9 (30.0–41.9)	41.0 (35.2–46.9)	30.2 (21.5–38.9)	36.0 (30.1–42.0)	40.0 (34.1–45.8)	30.1 (22.3–38.0)	35.4 (29.5–41.4)
Vitality (VAS)	—	62.3 (57.0–67.6)	62.8 (54.3–71.2)	61.3 (55.8–66.9)	57.8 (52.4–63.1)	56.2 (46.7–65.7)	58.6 (53.0–64.1)	56.0 (50.7–61.4)	59.3 (50.7–67.9)	57.4 (51.9–62.9)
Well-being (VAS)	—	63.0 (56.7–69.3)	63.5 (54.7–72.3)	63.0 (56.9–69.0)	61.5 (55.2–67.8)	59.0 (49.0–69.0)	62.1 (56.1–68.1)	58.5 (52.2–64.8)	64.0 (55.1–73.0)	60.5 (54.5–66.6)

Results from linear mixed models adjusted for individual’s baseline values. aMean, adjusted mean; CI, Confidence Intervall; BP, blood pressure; VAS, Visual analogue scale.

**Figure 7:**
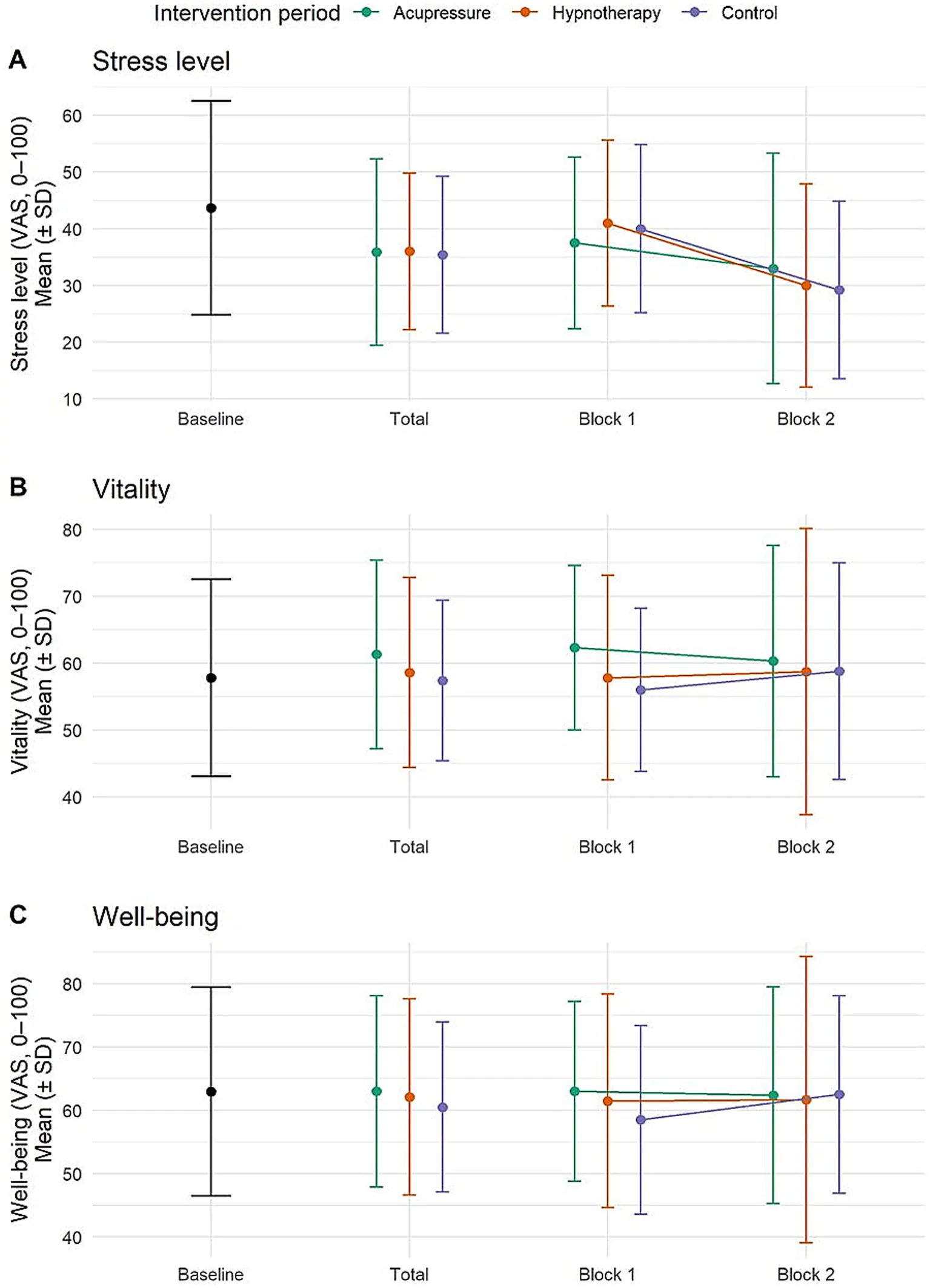
Baseline (mean of measurements during run-in period) and follow-up mean values (± one standard deviation) for VAS stress, vitality, and well-being **(A–C)**.

In the process of the study, mean perceived stress levels were lower compared to the run-in period (Figure 7A). However, stress values showed substantial variability, as reflected by high standard deviations. Overall, only minor differences were observed between periods and blocks. VAS for vitality showed slightly better values during the acupressure periods in both blocks. Adjusted mean values were 3 to 6 points higher in the acupressure periods than in the hypnosis or control periods (Tables 2, 3; Figure 7B,C).

### 3.6 Changes during the washout periods

Evaluation of the washout periods showed no consistent pattern indicative for carryover effects (Supplementary Figure 5). Nevertheless, the high variability of the outcome measures reduced statistical power, making it difficult to distinguish potential carryover effects from random fluctuations. Therefore, the absence of detectable carryover effects should be interpreted with caution.

### 3.7 Subgroup analyses

Men and women differed in the variability of BP outcomes: women exhibited standard deviation that were two- to threefold higher than those of men (Supplementary Table 3). Especially for systolic BP, women had higher variation in measurements compared to men (e.g., during hypnosis in the first block, mean (SD), 129.6 (18.6) vs. 128.2 (7.9)). However, no consistent differences in mean levels between women and men were observed.

The relatively small number of patients in the subgroup analysis limits our ability to draw firm conclusions regarding subgroup effects, especially for age (≥50/≤50 years) and grade of hypertension.

### 3.8 Adverse events

During the study period one severe unexpected event happened, which was not related to one of the study interventions. Five patients reported side effects of which all complaints were minor and temporary. During acupressure periods four patients documented side effects like temporary short-term pain and sensitivity disorders in different areas. Only one patient reported a single episode of restlessness during hypnosis with an urge to move during the hypnosis session.

## 4 Discussion

### 4.1 Summary

The results of this study showed a good feasibility of N-of-1 studies, reflected by high adherence to the self-administered study interventions, including weekly changes between interventions with washout periods. Only few mild and temporary therapy related adverse events occurred, and more in acupressure compared to hypnosis. Looking at the results of the individual N-of-1 trials, three patients showed lower BP values during acupressure and/or hypnosis periods. However, it is unclear if this is related to the specific intervention or the ability for relaxation. Furthermore, this was a pilot feasibility study. While it was able to demonstrate feasibility the potential effects of the interventions could not be proven. Nevertheless, the results underscore the potential of N-of-1 studies for individual patients and their possible relevance to personalized clinical decision-making.

No effects of the interventions could be observed at the group level in the meta-analysis of individual patient data, neither for cardiovascular outcomes nor for subjectively perceived stress, vitality and well-being.

### 4.2 Strengths

This study was the first series of N-of-1 trials to investigate the feasibility and potential individual effects of acupuncture and hypnosis in arterial hypertension. Study procedures and analyses were defined in detail in advance. All patients could be recruited quickly, and the study proceeded smoothly and routinely. Patients largely followed the given instructions for measurement and documentation of outcomes, there was only one drop out, which was not related to the study.

After the study was completed, patients were informed of the results in writing and in person. Because the results were discussed between the study physicians and the patients after the study ended, it was possible to focus on individual results and aspects of hypertension treatment in general—primarily based on individual meta-analyses. In addition, the results of the mixed-methods design provided further insights into the effectiveness and feasibility of the intervention.

### 4.3 Limitations

There are several factors that limit the validity of our study’s results. The interventions and cardiovascular measurements were performed by the patients themselves at home. Although the patients received detailed instructions and information on how to perform the interventions and measure the cardiovascular parameters during their first visit to the study center, and had the opportunity to contact the study secretariat at any time with questions, implementation at home was not consistently monitored as part of this pragmatic study. Also, there was no further calibration of the BP devices during the study. Therefore, the accuracy of the cardiovascular results could be called into question. Additionally, this limitation may also have contributed to the high variability of the measured values.

Furthermore, the study procedures did not specify an exact time for measurements. As blood pressure may fluctuate over the course of the day, for example due to chronobiological modulation, the absence of a defined measurement time point may have contributed to greater variability and reduced validity of the measured values.

It cannot be ruled out that a 24-h ambulatory BP measurement (ABPM) would have shown different and more precise results, especially regarding the relationship between measuring timepoint and intervention. The decision to use a simple, standardized and proven method of measuring cardiovascular parameters, namely home-based BP measurement using an upper arm cuff instead of an ABPM, was primarily made for pragmatic reasons. It corresponds to the measurement behavior of most patients and, when performed in a standardized manner, is relatively accurate and less stressful than ABPM and is therefore recommended in guidelines (2, 30). In the future, wearables will become a suitable and frequently used possibility for long term BP measurements.

Also, we chose a standardized rather than individualized acupressure and hypnosis approach, which may differ from the highly individualized therapeutic approach patients may receive in clinical reality with therapists and influence the effects of an intervention. We decided this way because we wanted to make the interventions as feasible as possible for the patients in the frame of self-applied treatment.

Other limitations could include the duration, intensity, and number of repetitions of the treatment and washout phases, which were also determined primarily for reasons of feasibility. Our analysis of washout periods showed no consistent pattern indicative for carryover effects. These results have to be taken with caution, again because of the large variability of outcome measures. To obtain meaningful results from an N-of-1 RCT, ideally at least five blocks should be included (31). Also, mind–body interventions may need a longer practice period to show relevant effects on BP. However, in our study, a longer study duration or a more intensive therapy might have reduced adherence and compliance. Therefore, the study duration was limited to what was considered a feasible time frame.

Another limitation was the lack of blinding and sham controls of the interventions, so that specificity of treatment could not be estimated.

Assuming individually different responses to hypnosis and acupressure, we decided to conduct this pragmatic series of N-of-1 trials. The prospective crossover single-patient study design allows precise and controlled assessment of intra-individual treatment effects in chronic, stable conditions such as arterial hypertension (32, 33) with patient-related and objective outcomes (28). The aggregation of the data from the individual studies in a meta-analysis enables to show the group benefit even with a smaller number of cases (34). However, in our study the sample size was relatively low and estimated under feasibility aspects. BP is characterized by a high variability over a 24-h period; accordingly, substantial variability was observed in our study at both the between-subject and within-subject (intra-individual) levels. To address this issue, a larger number of patients and blocks would be required to minimize the impact of a high standard deviation and potential confounding factors. A larger sample size may have contributed to a higher statistical power and a better external validity of results including more options for subgroup analysis.

The N-of-1 RCT is a relatively new design, and very few N-of-1 RCTs have been conducted in medicine to date (35, 36). Various reasons led us to choose the series of N-of-1 study design. N-of-1 RCTs are particularly important as this design can accommodate individual patient conditions and preferences, allowing to identify the best of two or more different interventions for the individual (31). Nikles et al. (37) were able to show that patients who underwent an N-of-1 RCT had an increased sense of control over health decisions. Greenfield et al. were even able to show that active engagement can improve health status. The N-of 1 procedure encourages patients to actively engage with their health status, which potentially leads to a better understanding and awareness of their health status (38).

However, this design has some requirements due to the crossover of intervention and control periods. It can be challenging to correctly estimate the expected duration of the lasting effect of the interventions in order to determine the length of the washout period. Interventions with very long-term effects are unsuitable for such a design, as the effects of different interventions can no longer be distinguished due to carryover effects (31). As very little is known about the overall duration of the effects of acupressure and hypnosis on hypertension, the estimation of the washout period was only possible to a limited extent.

Our study showed good feasibility due to the professional study team in the background and the high patient compliance. The comparison of individual-level effects of adjunctive acupressure or hypnosis versus RC identified three patients who showed reduced BP during acupressure and/or hypnotherapy. This brings up the question, what kind of individual conditions may have influenced the effects of hypnosis and acupressure. Besides distinct aspects of medical history, effects may be also dependent on individual suggestibility and possibilities for a relaxation response in general. Both interventions in our study targeted a relaxing effect, since its relevance for hypertension is known. From a clinical perspective it may be interesting if it would be possible to estimate patients´ response by testing for suggestibility and relaxation response (39). This should be the subject of further research and consideration in medical practice.

However, most individual-level analyses and the aggregated data did not show relevant changes of outcomes related to the intervention. Beneath the size of the sample, the way of measurement of cardiovascular outcomes and the performance of self-administered interventions, the potential role of an effective anti-hypertensive medication remains unclear.

The latter likely resulted in baseline blood pressure readings falling within the normal range or reaching Stage 1, which could limit the potential for additional effects of acupressure or hypnosis as adjunctive therapies. By comparison, for example, the study by Lin et al. on the short-term effects of acupressure on the Liver 3 (TaiChong) point included patients with significantly higher baseline readings (165/96 mmHg) (9). In this study, acupressure resulted in a significant reduction—particularly in systolic blood pressure—15 and 30 min after treatment compared to the sham control. The observed effects of acupressure were independent of the antihypertensive medications taken by 45% of the patients.

In our study it might have been possible to detect short-term effects of the interventions, if the timing of measurements and interventions had been arranged such that assessments were consistently conducted immediately after acupressure or hypnosis. In contrast to other studies, with a focus on short term effects of acupressure (10, 40) or hypnosis (19) on BP, we deliberately chose not to do this in order to be able to observe medium-term effects. From a clinical perspective, these are more relevant than short-term effects for the treatment of chronic conditions such as hypertension.

Taken together, several of the limitations mentioned here were linked to decisions we made to improve the feasibility of the study and make it easier for patients to adhere to the required study procedures, while feasibility was the primary aim of our pilot study.

Future research should investigate whether longer intervention periods and a longer study duration are feasible and can lead to mid- or long- term effects on the cardiovascular system. Qualitative research might be used to get more insights into this question. The series of *N* = 1 studies is a design that should be used more often in certain conditions and treatments, however to our experience in this trial it requires very precise planning and a good knowledge of the study interventions used. The results of our study cannot be generalized due to several limitations and the pilot character of the study, but it may deliver a valuable basis for further research.

## 5 Conclusion

This series of N-of-1 RCTs investigating self-administered acupressure and hypnosis in hypertension was found to be feasible, as patients showed a good adherence to the interventions. No relevant changes in outcome parameters, including blood pressure (BP), were observed in the overall sample, although selected patients showed individual changes.

However, owing to the pilot design, the small sample size, and the limitations discussed above, no conclusions can be drawn regarding the effectiveness of the interventions or potential BP benefits in individual patients. Nevertheless, these findings provide a valuable basis for future research.

Future studies should further standardize and validate measurement and treatment procedures and account for individual factors like suggestibility and expectation.

## Data Availability

The raw data supporting the conclusions of this article will be made available by the authors, without undue reservation.
