# Supplementary material for: Acupressure and hypnosis in arterial hypertension: a series of N-of-1 trials
Source: Front Med (Lausanne). 2026 Aug 17;13:1871793. doi: 10.3389/fmed.2026.1871793 (PMC13526541; doi:10.3389/fmed.2026.1871793)

Supplemental Material for:

Non-medical Therapies in Hypertension: A series of N-of-1 trials on Acupressure and Hypnosis

Miriam Ortiz, Max Marco Schreiner, Nicolas Volz, Tatjana Tissen-Diabaté, Eva Jansen, Julia Siewert, Michael Teut, Benno Brinkhaus

This document contains supplemental information supporting the results presented in the main manuscript, including extended methods, additional analyses, and supplemental figures and tables.

Table of contents

1. Figures Meta-analysis	Fig. 1a: Meta-analysis pulse rate hypnotherapy vs. control (routine care alone)<br>Fig. 1b: Meta-analysis pulse rate pressure acupressure vs. control (routine care alone)<br>Fig. 1c: Meta-analysis pulse rate acupressure vs. hypnotherapy<br>Fig. 2a: Meta-analysis VAS well-being hypnotherapy vs. control (routine care alone)<br>Fig. 2b: Meta-analysis VAS well-being acupressure vs. control (routine care alone)<br>Fig. 2c: Meta-analysis VAS well-being acupressure vs. hypnotherapy<br>Fig. 3a: Meta-analysis VAS vitality hypnotherapy vs. control (routine care alone)<br>Fig. 3b: Meta-analysis VAS vitality acupressure vs. control (routine care alone)<br>Fig. 3c: Meta-analysis VAS vitality acupressure vs. hypnotherapy<br>Fig. 4a: Meta-analysis VAS stress hypnotherapy vs. control (routine care alone)<br>Fig. 4b: Meta-analysis VAS stress acupressure vs. control (routine care alone)<br>Fig. 4c: Meta-analysis VAS stress acupressure vs. hypnotherapy
2. Washout phases	Fig. 5: Individual trajectories of systolic and diastolic blood pressure during the final day of the intervention period and the subsequent two-day washout phase.
3. Measure-ment times	Fig. 6: Timing of home blood pressure measurements over the 6-week study period
4. Tables	Table 1: Antihypertensive Medication<br>Table 2: Subgroup Analyses for systolic and diastolic blood pressure, morning and evening measurements, across the two blocks, by grade of hypertension, sex and age categories (≤50/>50). Descriptive statistics by treatment period.<br>Table 3 subgroup analysis
5. Information for patients' results	Text and Figures (original version in German)

1. Figures Meta-analyses

Fig. 1a: Meta-analysis pulse rate hypnotherapy vs. control (routine care alone)

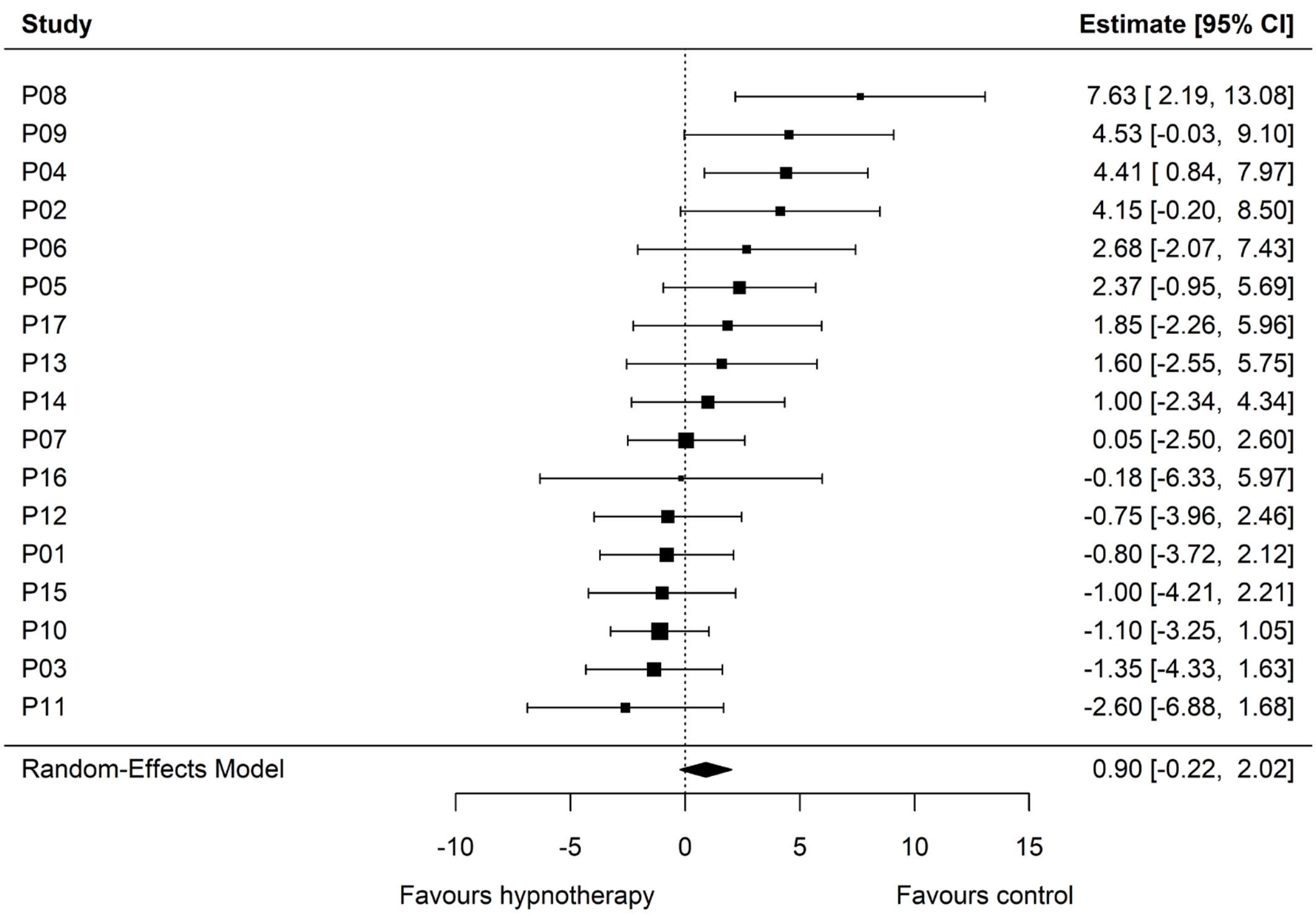

Fig. 1b: Meta-analysis pulse rate pressure acupressure vs. control (routine care alone)

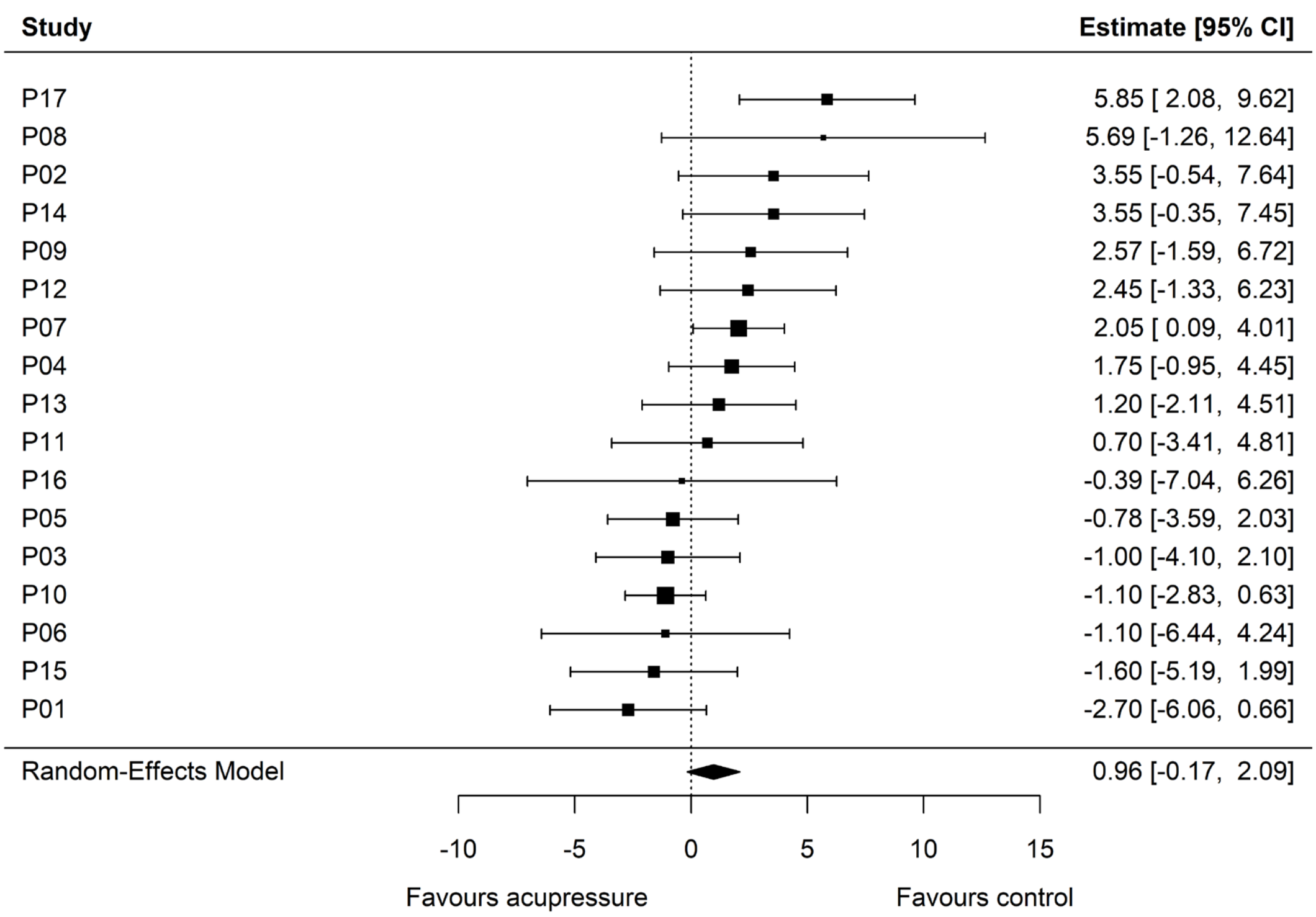

Fig. 1c: Meta-analysis pulse rate acupressure vs. hypnotherapy

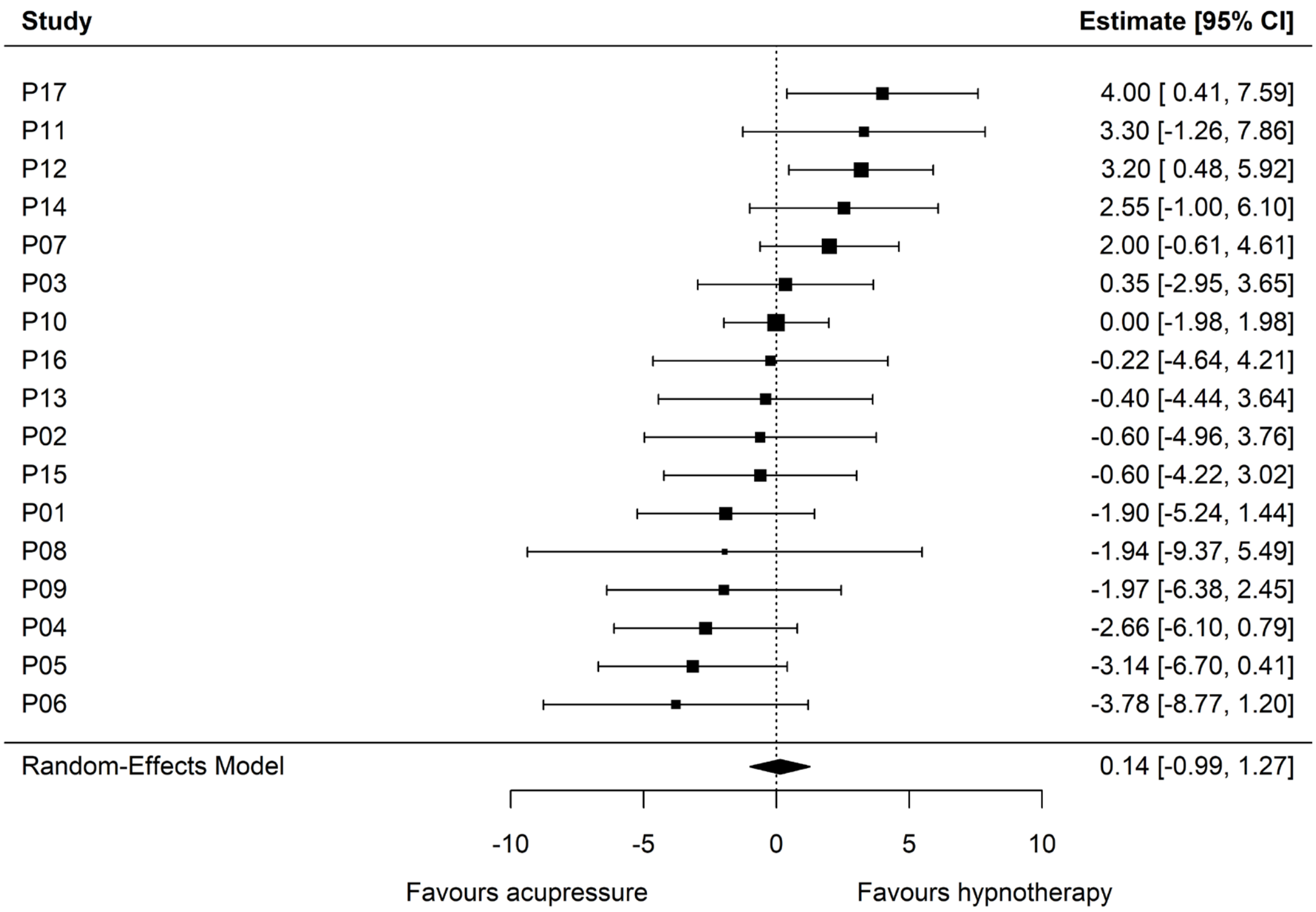

Fig. 2a: Meta-analysis VAS well-being hypnotherapy vs. control (routine care alone)

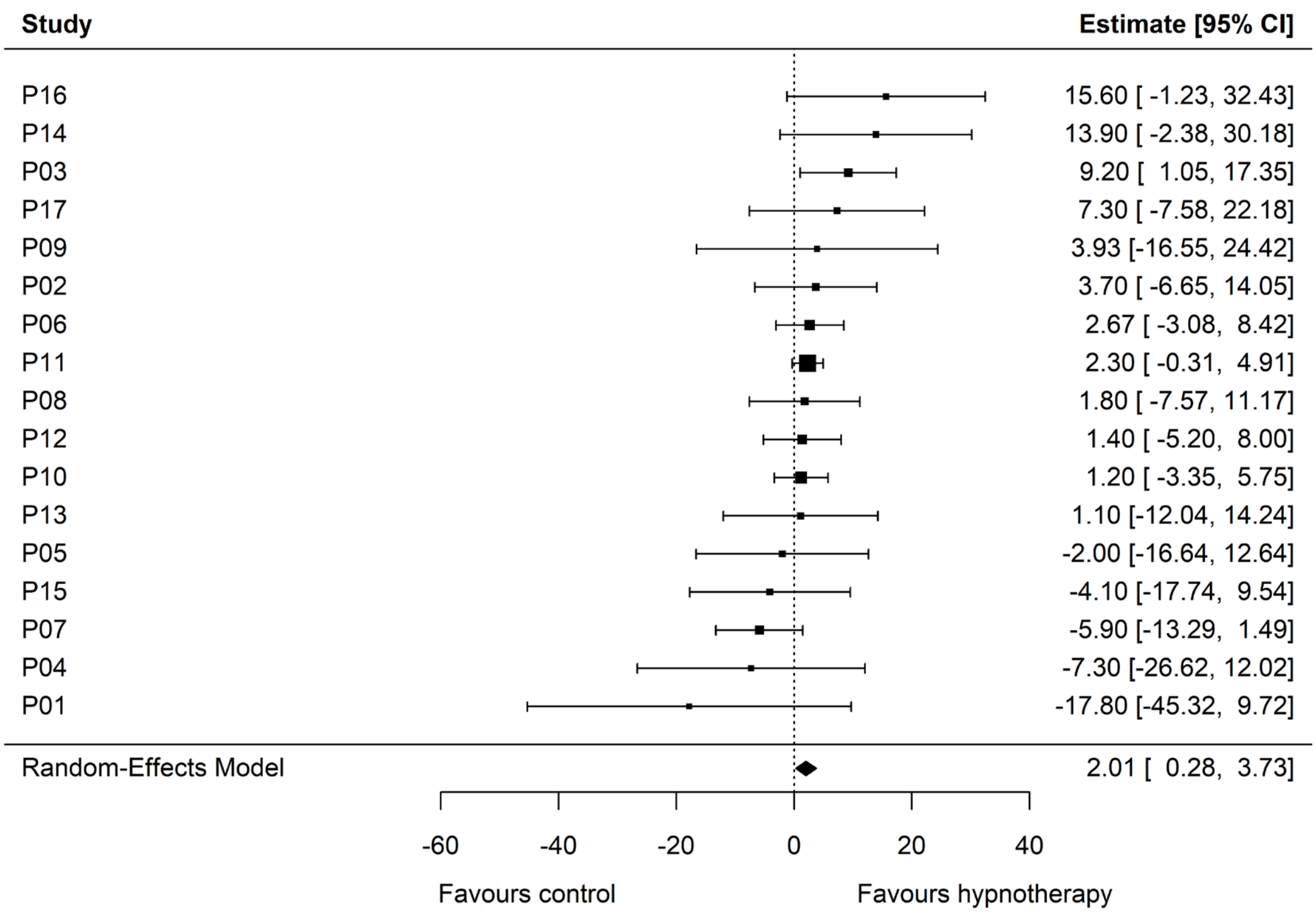

Fig. 2b: Meta-analysis VAS well-being acupressure vs. control (routine care alone)

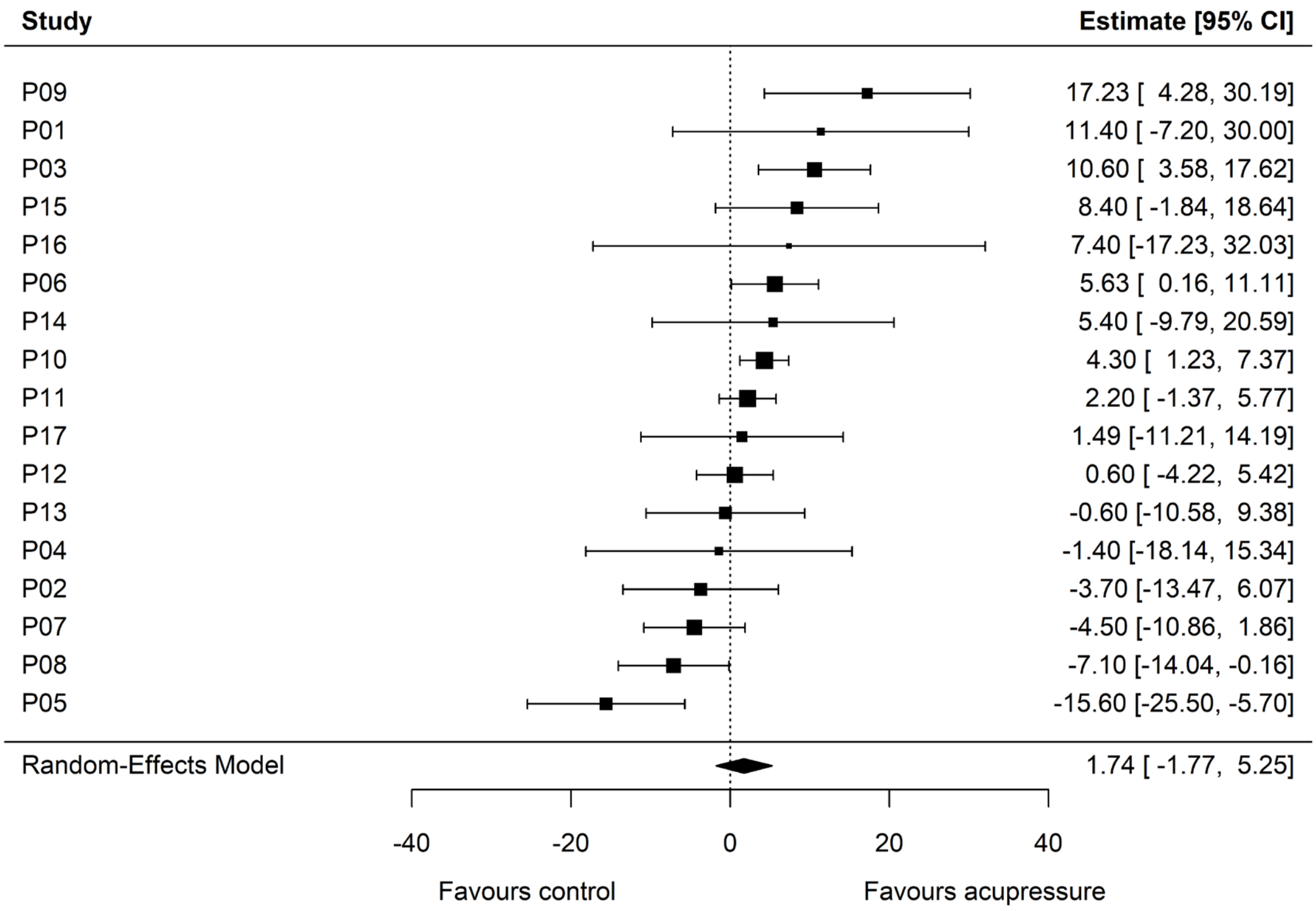

Fig. 2c: Meta-analysis VAS well-being acupressure vs. hypnotherapy

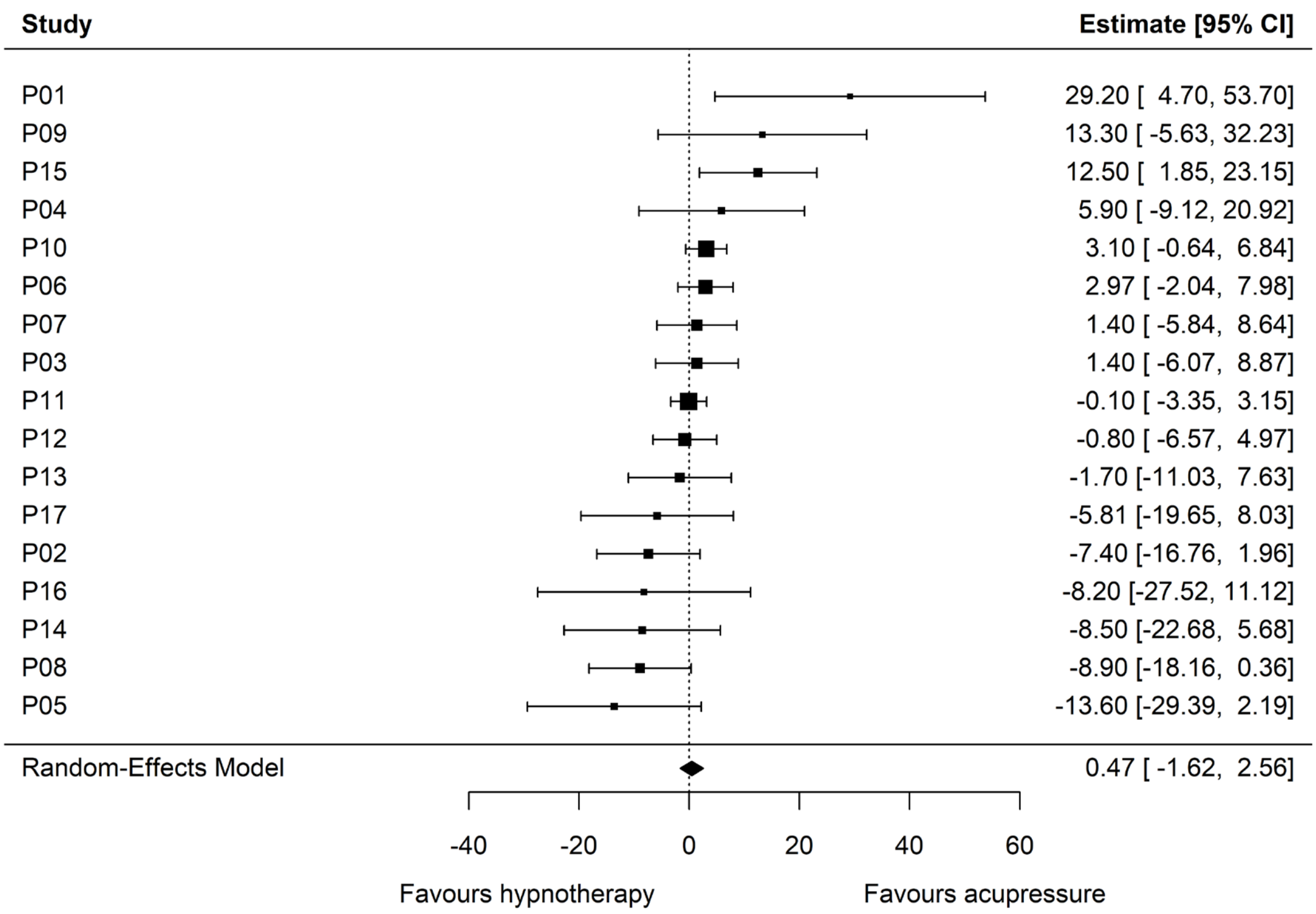

Fig. 3a: Meta-analysis VAS vitality hypnotherapy vs. control (routine care alone)

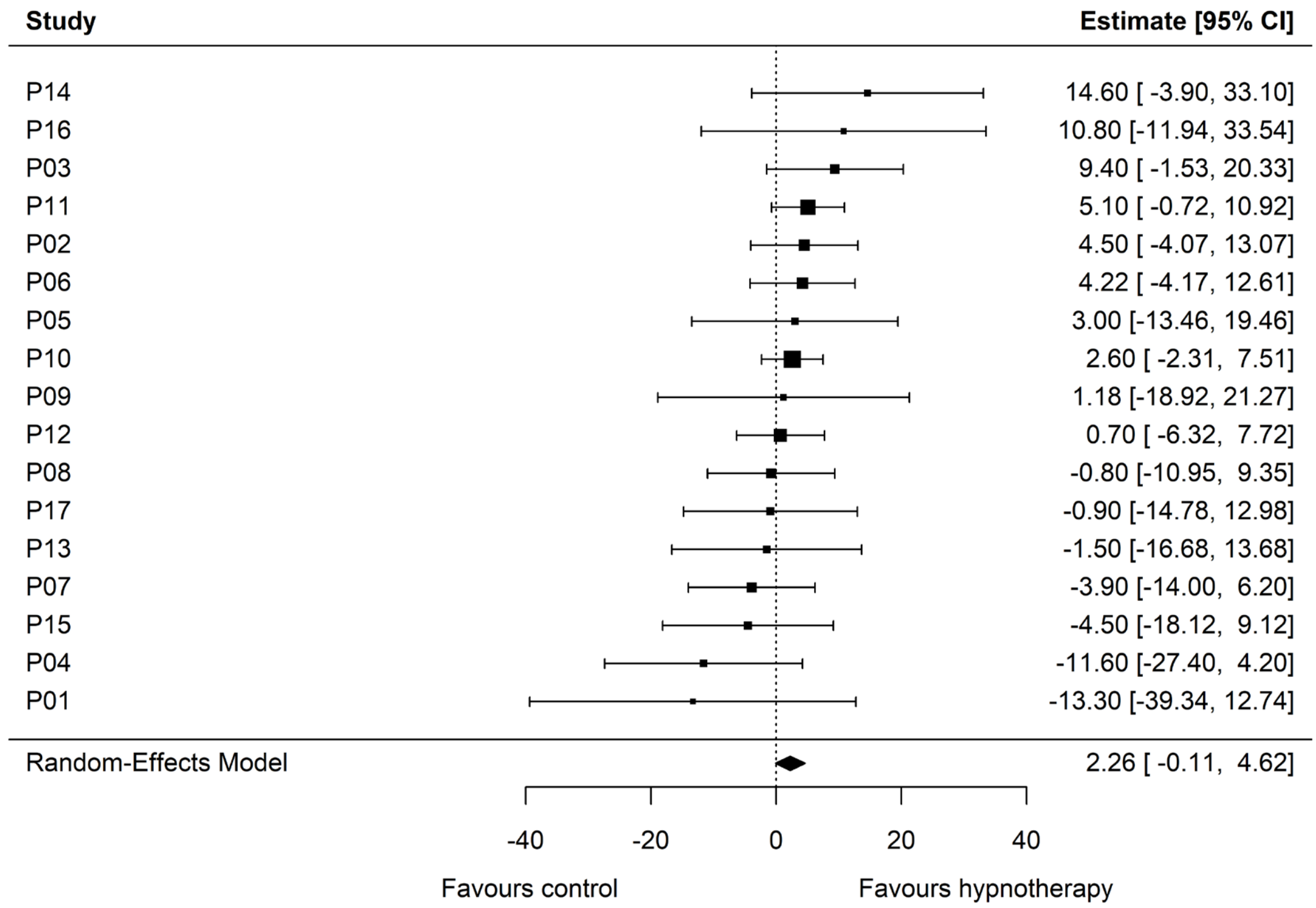

Fig. 3b: Meta-analysis VAS vitality acupressure vs. control (routine care alone)

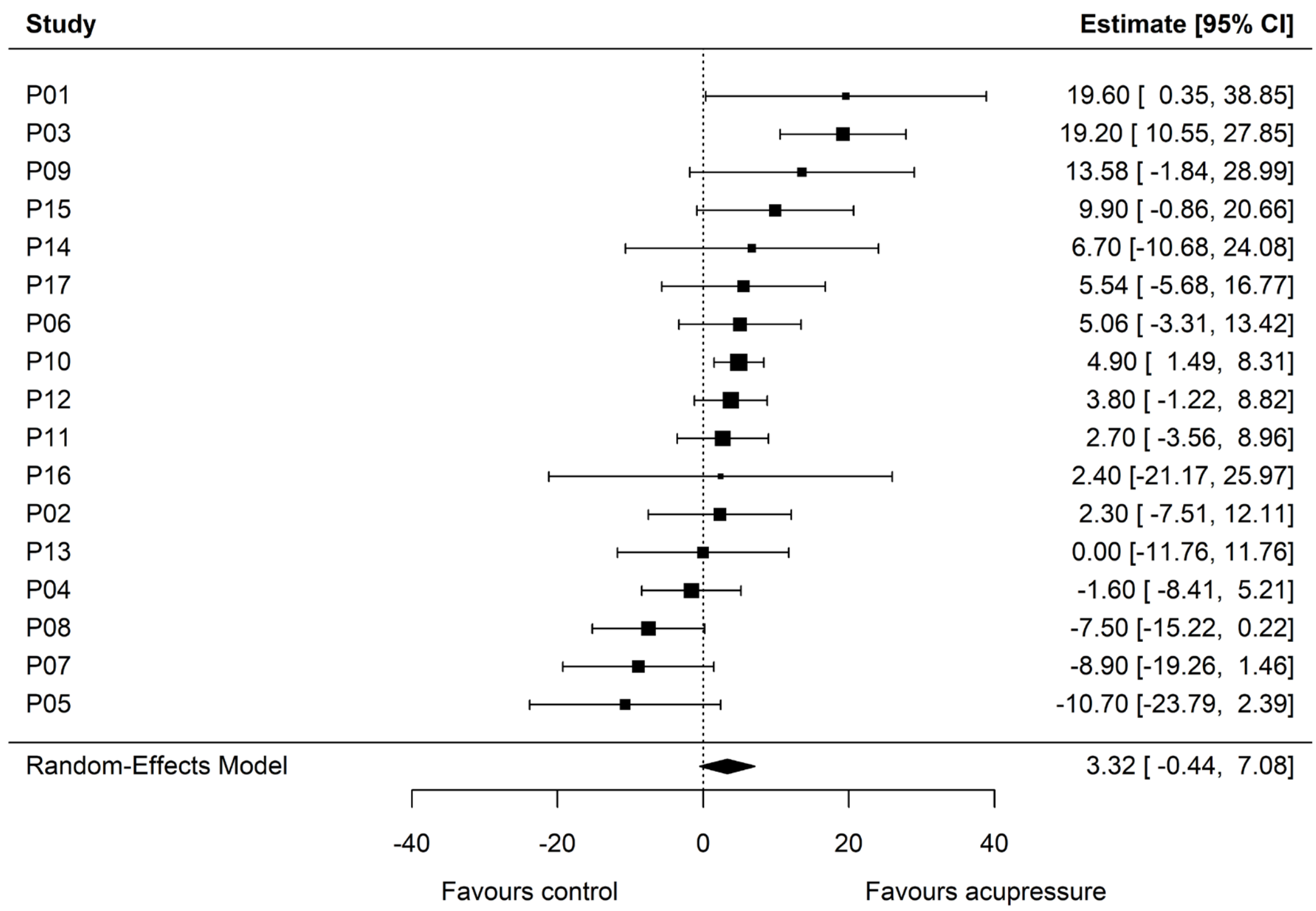

Fig. 3c: Meta-analysis VAS vitality acupressure vs. hypnotherapy

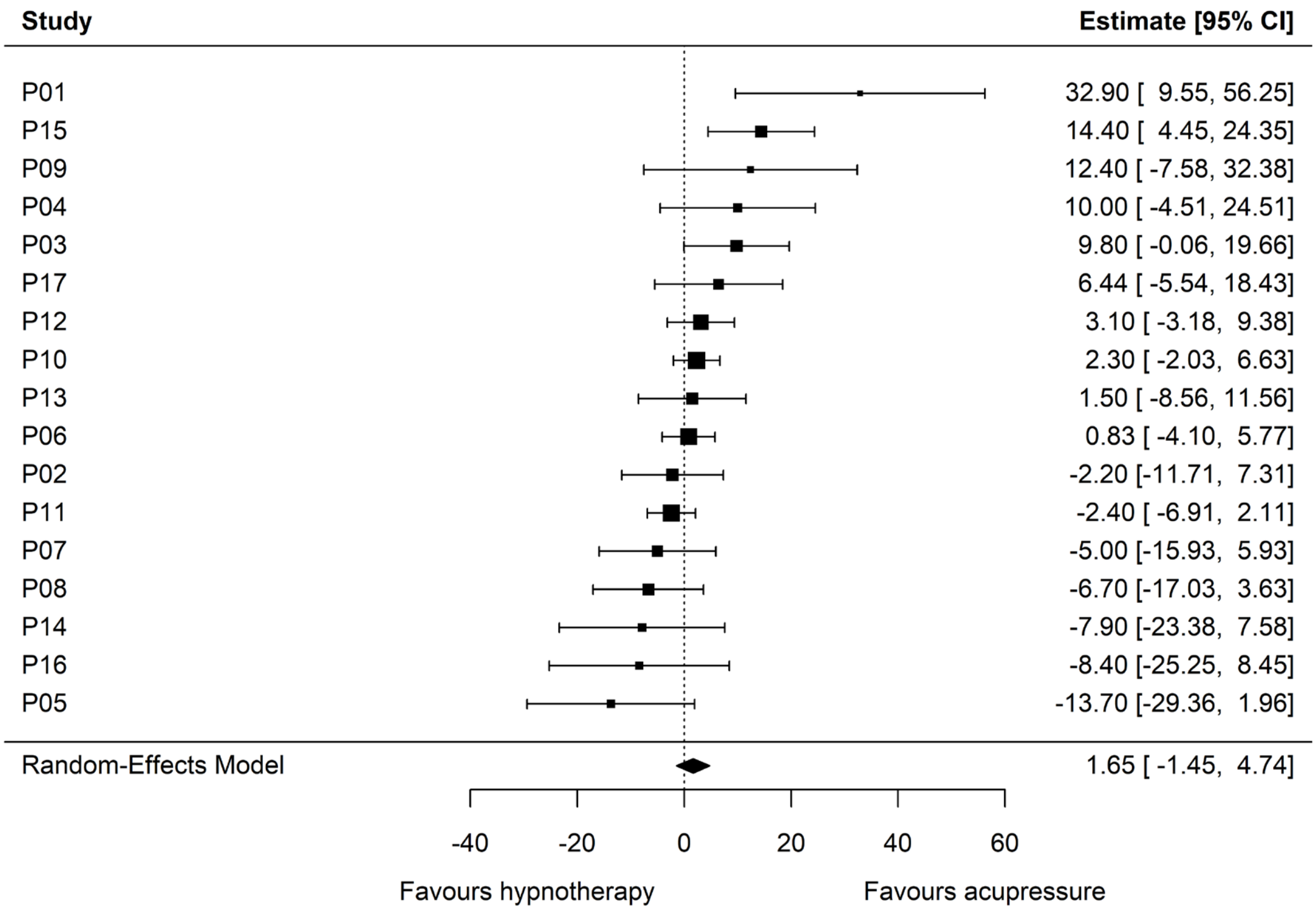

Fig. 4a: Meta-analysis VAS stress hypnotherapy vs. control (routine care alone)

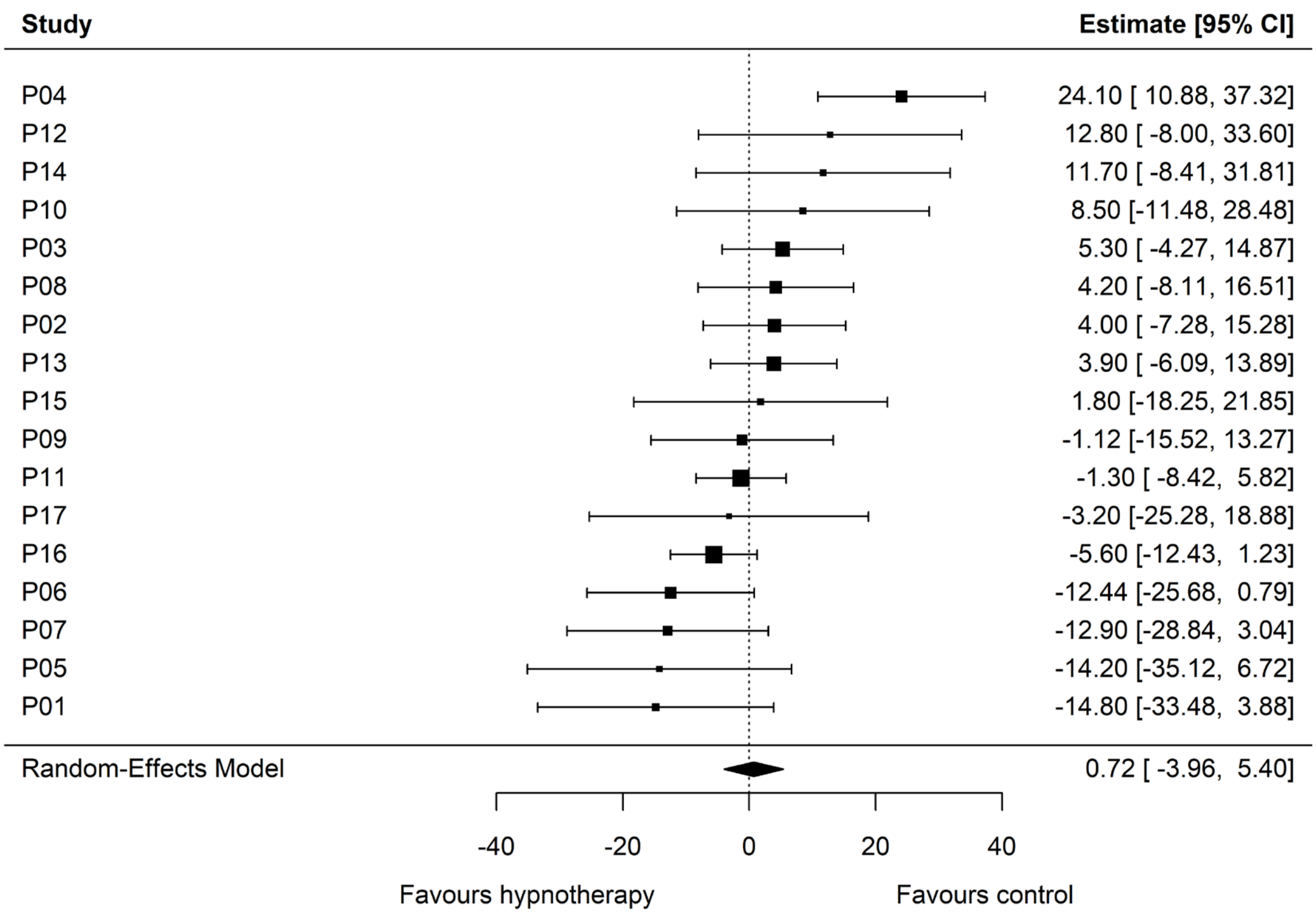

Fig. 4b: Meta-analysis VAS stress acupressure vs. c control (routine care alone)

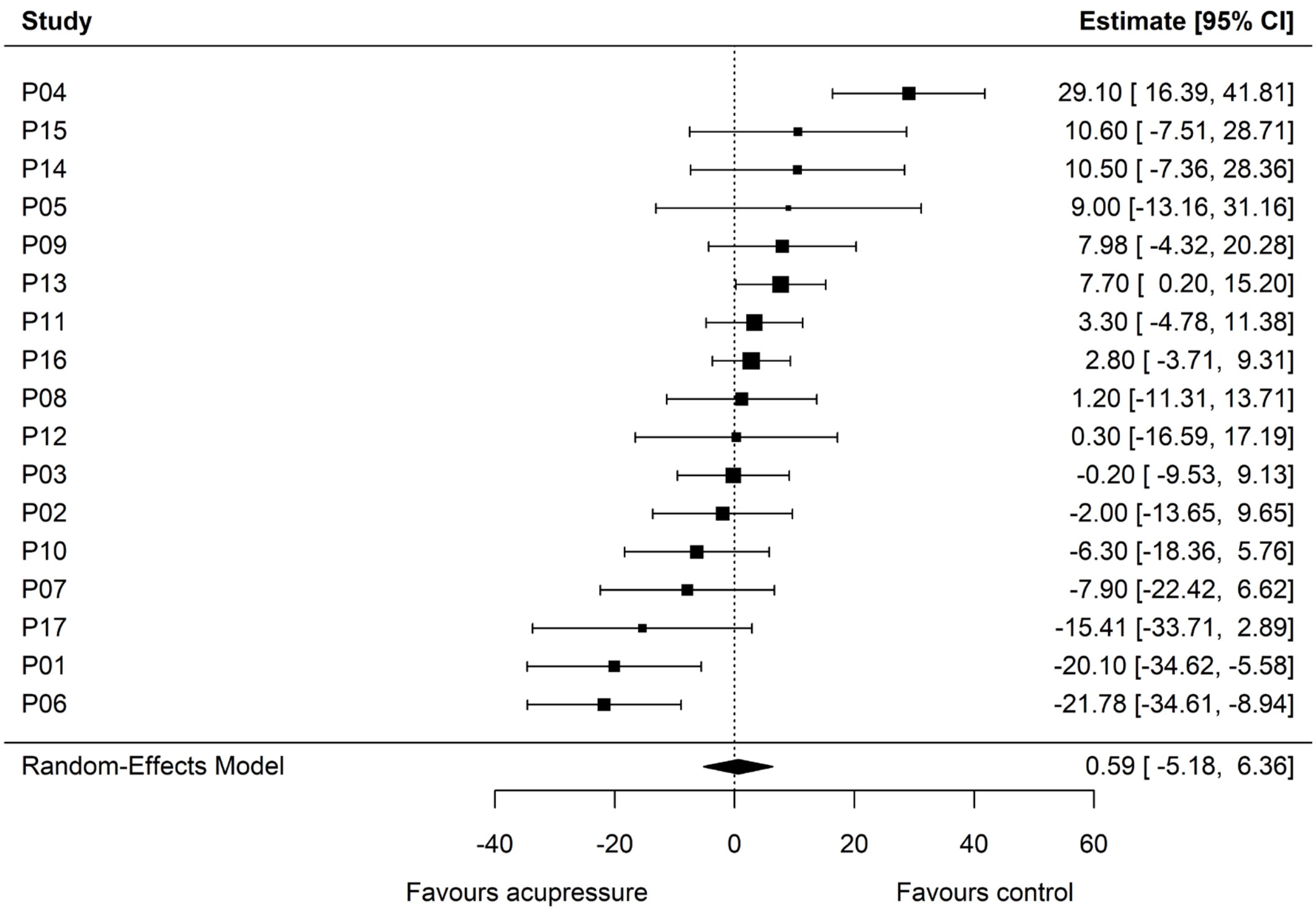

Fig. 4c: Meta-analysis VAS stress acupressure vs. hypnotherapy

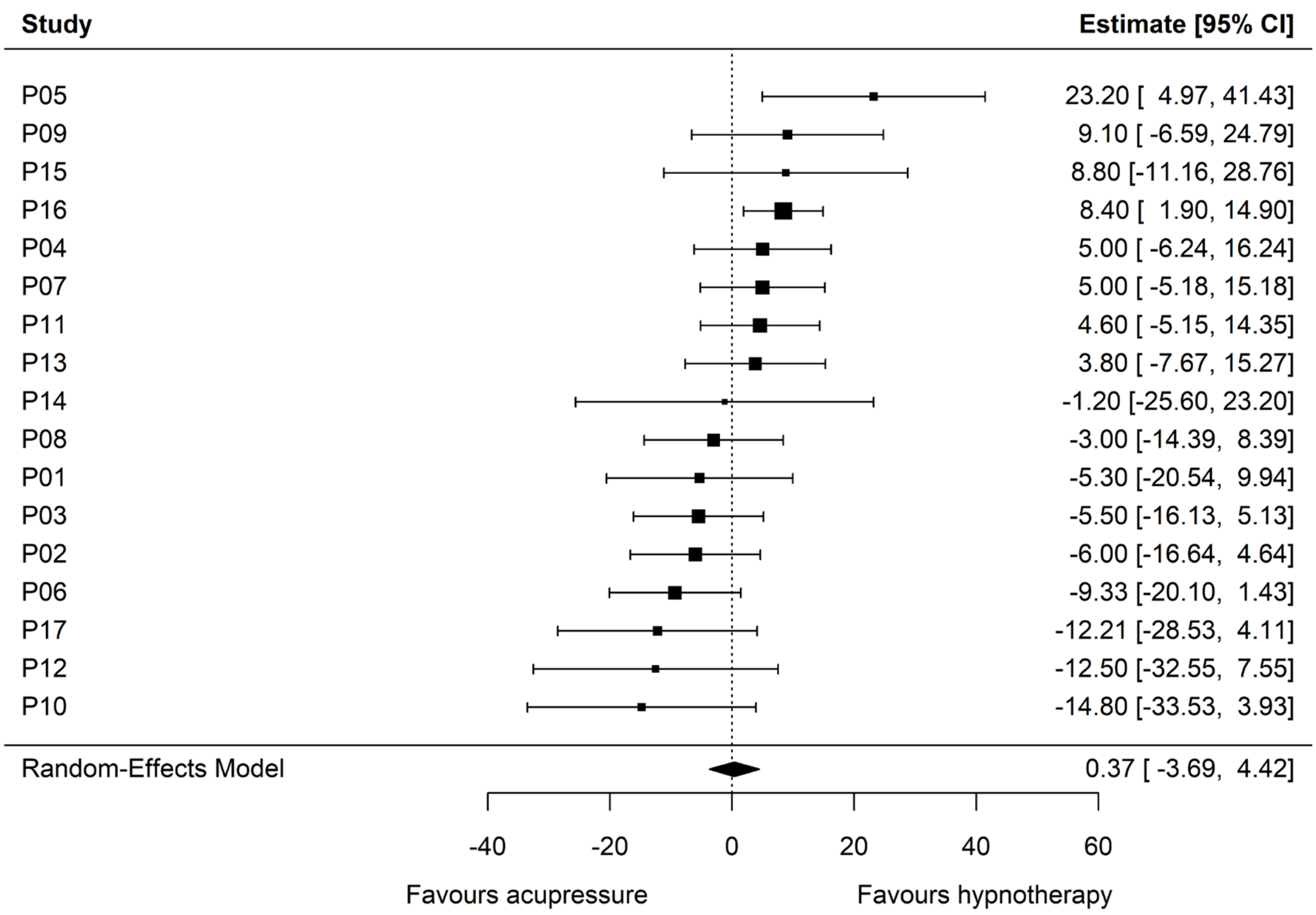

2. Washout periods

Fig. 5: Individual trajectories of systolic and diastolic blood pressure during the final day of the intervention period and the subsequent two-day washout.

Systolic blood pressure is shown in the upper panels and diastolic blood pressure in the lower panels, with morning and evening measurements presented separately. Results are displayed by block and intervention. Each coloured line represents an individual participant, while the black solid line depicts the overall linear regression trend across all individuals.

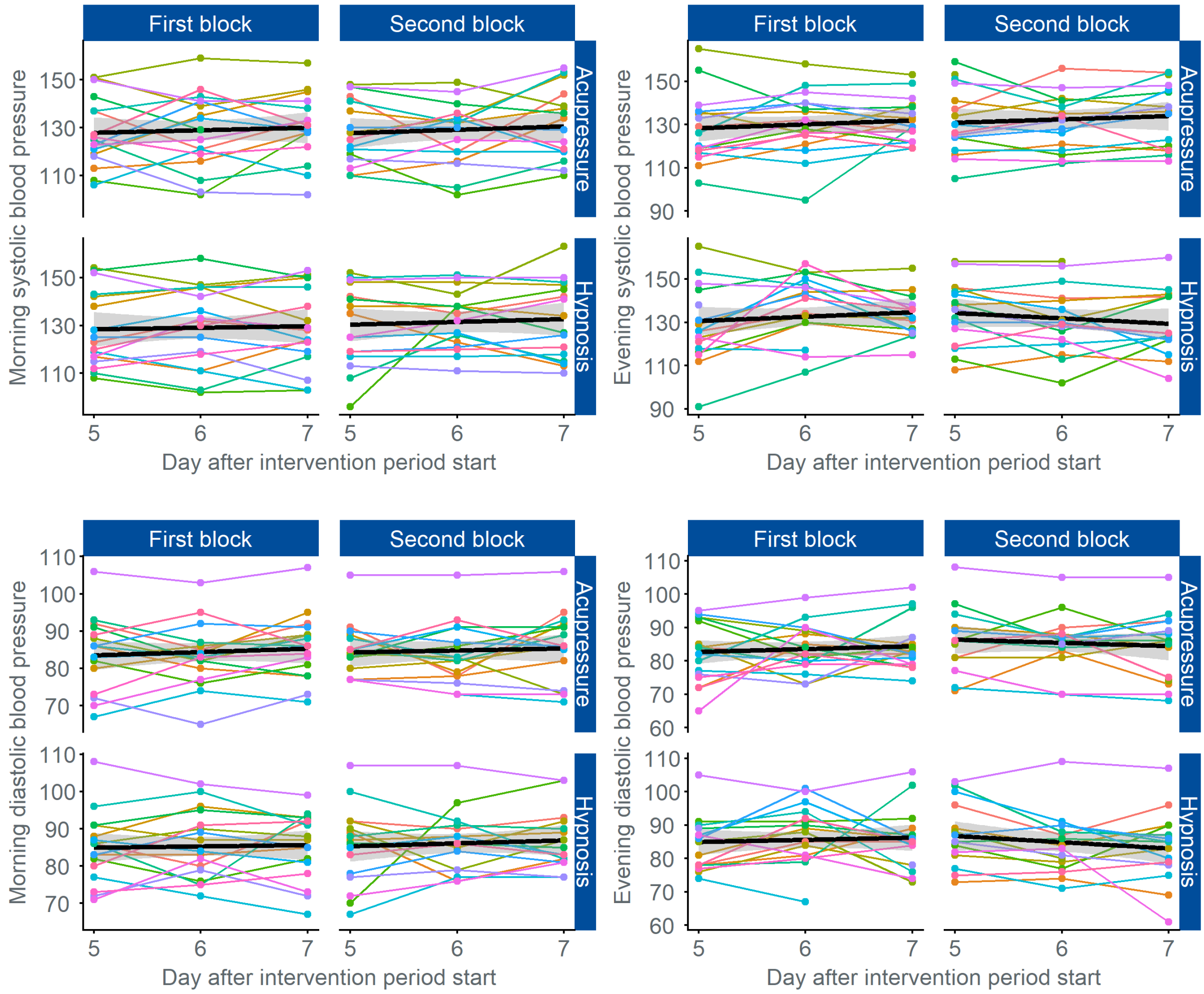

3. Measurement times

Fig. 6: Timing of home blood pressure measurements over the 6-week study period. Each point represents a single blood pressure measurement, with the measurement time shown on the y-axis and study week on the x-axis. Panels display morning and evening measurements separately. Points are coloured by sex (female, male). The solid lines represent participant-specific weighted polynomial regression (LOESS, locally estimated scatterplot smoothing) curves, illustrating individual measurement patterns over time.

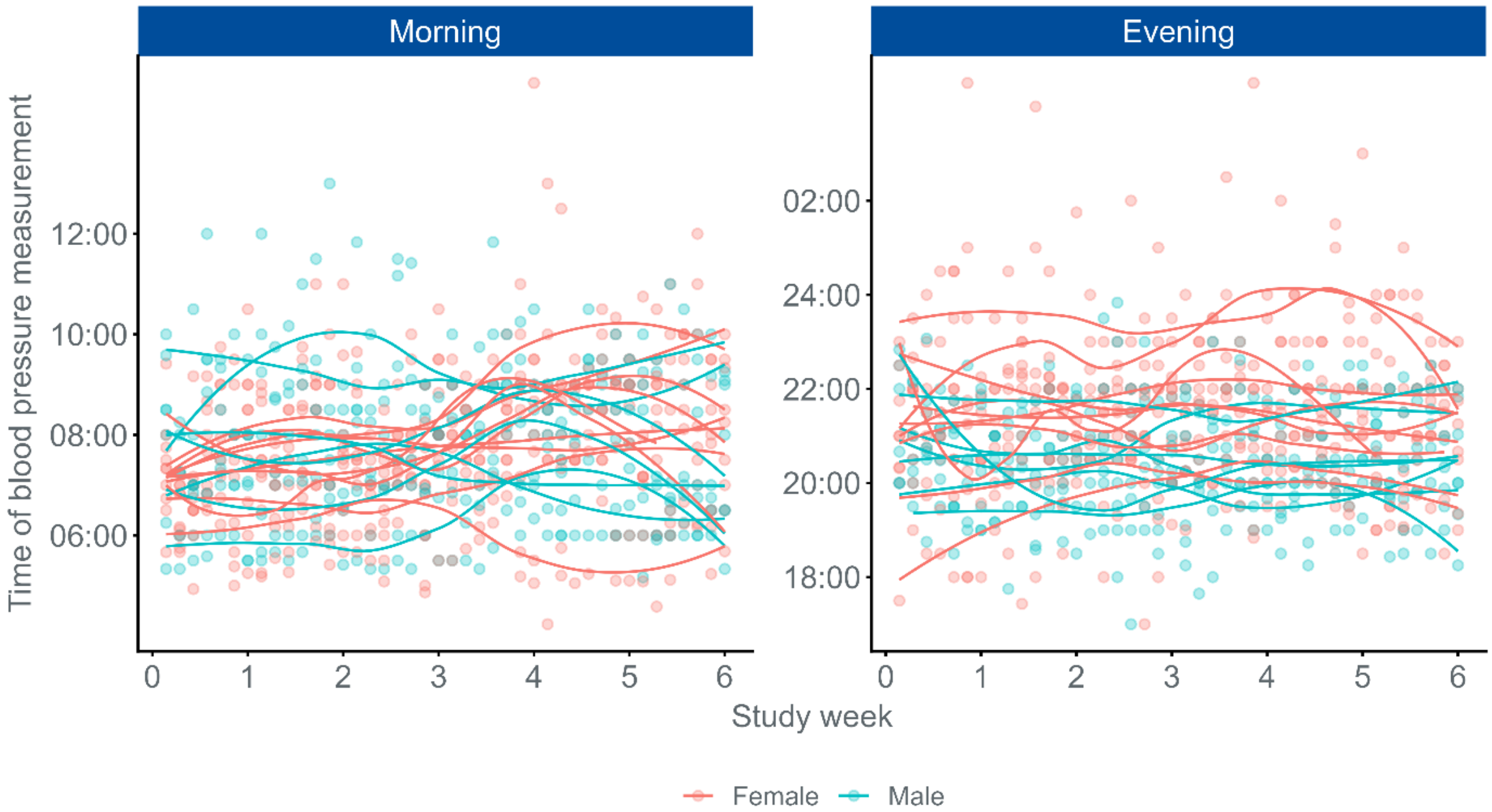

4. Tables

Tab. 1: Antihypertensive Medication

**Patients**	**Medication 1**	**Medication 2**
P01	Candesartan	-
P02	Ramipril	HCT
P03	-	-
P04	Candesartan (AT 1-Rezeptorantagonist	
P05	-	-
P06	Candecor	-
P07	Ramipril	Amlodipin
P08	Ramipril	-
P09	Candesartan	-
P10	Amlodipin	Candesartan
P11	Amlodipin	Fludex (Diureticum)
P12	Coveram (ACE+Ca Antag.)	-
P13	Ramipril	Metoprolol
P14	Vocado	-
P15	Candesartan	Metoprolol
P16	Candesartan	-
P17	Candecor	-

Tab. 2: Baseline characteristics of patients who responded to acupressure and/or hypnosis

**Variable**	**P02**	**P11**	**P17**
Age	62	59	55
Sex	female	male	male
BMI	37.9	28.4	40
Years with hypertension	17	9	23
Highest education	university	university	university
Treatment expectation hypnosis	effective	not very	effective
acupressure	effective	not very	very
Medication	Ramipril	Amlodipin	Candecor
Restricted salt consumption	yes	yes	yes
Nicotin consumption	No	No	No
MET-Minutes total	960	5240	900
Systolic blood pressure (run-in period)	128.2 (10.0)	127.6 (7.4)	133.1 (6.0)
Diastolic blood pressure (run-in period)	86.1 (5.8)	85.9 (4.8)	89.9 (5.7)
Pulse rate (run-in period)	69.0 (2.9)	71.0 (5.6)	87.9 (6.3)

Tab. 3: Subgroup Analyses for systolic and diastolic blood pressure, morning and evening measurements, across the two blocks, by grade of hypertension, sex and age categories (≤50/>50). Descriptive statistics by treatment period. If fewer than three observations were available, no summary statistics were calculated and individual values are presented instead.

			**Systolic blood pressure**			**Diastolic blood pressure**		
**daytime cardiovascular measurements**	**Subgroup**	**Subgroup value**	**Acupressure group Mean (SD)**	**Hypnosis group Mean (SD)**	**Control group Mean (SD)**	**Acupressure group Mean (SD)**	**Hypnosis group Mean (SD)**	**Control group Mean (SD)**
morning	Blood pressure	Grade 1 (n=15)	125.6 (9.5)	126.2 (10.6)	128.1 (10.7)	83.4 (5.8)	83.7 (5.6)	84.8 (5.9)
		Grade 2 (n=2)	145.1; 155	150.3; 157.3	148.5; 157.1	87; 104.9	86; 104.6	86.7; 103.6
evening	Blood pressure	Grade 1 (n=15)	129.6 (9.8)	129.3 (9.9)	129.3 (9.1)	83.6 (6.3)	84.1 (5.7)	84.0 (6.0)
		Grade 2 (n=2)	149.4; 159.8	152.9; 161.4	150.9; 156	86.1; 101.8	84.7; 103.6	85.4; 100.5
morning	Sex	Female (n=10)	128.4 (15.1)	130.2 (16.5)	131.7 (15.3)	84.4 (9.3)	85.4 (9.2)	86.7 (9.0)
		Male (n=7)	128.5 (7.4)	128.5 (9.1)	130.0 (9.9)	85.4 (4.7)	84.6 (3.7)	85.1 (3.5)
evening	Sex	Female (n=10)	132.4 (15.5)	131.6 (16.6)	131.7 (14.5)	84.4 (8.8)	84.6 (9.1)	84.5 (8.5)
		Male (n=7)	132.8 (7.4)	134.1 (6.5)	132.8 (7.2)	85.5 (5.3)	86.2 (3.0)	85.7 (4.3)
morning	Age	Age ≤50 (n=4)	127.2 (13.9)	128.6 (15.9)	130.2 (17.4)	84.4 (5.7)	86.1 (5.5)	86.9 (5.8)
		Age > 50 (n=13)	128.8 (12.2)	129.8 (13.5)	131.2 (12.2)	85.0 (8.2)	84.8 (7.9)	85.8 (7.7)
evening	Age	Age ≤50 (n=4)	137.1 (8.2)	136.1 (7.3)	133.8 (9.5)	87.5 (6.1)	88.3 (4.3)	86.4 (7.1)
		Age > 50 (n=13)	131.1 (13.5)	131.5 (14.6)	131.6 (12.6)	84.0 (7.7)	84.3 (7.7)	84.6 (7.1)

5. Information for patients’ results (example in German)

Auswertungen der ACUHyp-Studie

Hochschulambulanz für Naturheilkunde und Prävention
Institut für Sozialmedizin Epidemiologie und Gesundheitsökonomie
der Charité – Universitätsmedizin Berlin

1. Ihre persönlichen Ergebnisse für den Blutdruck

In der Abbildung 1 sehen Sie Ihre persönlichen Blutdruckwerte im Verlauf der gesamten Studie. Rechts neben der Abbildung finden Sie die Erklärung der farblichen Markierungen und Linien.

Abbildung 1: Ihre Blutdruckwerte im Verlauf

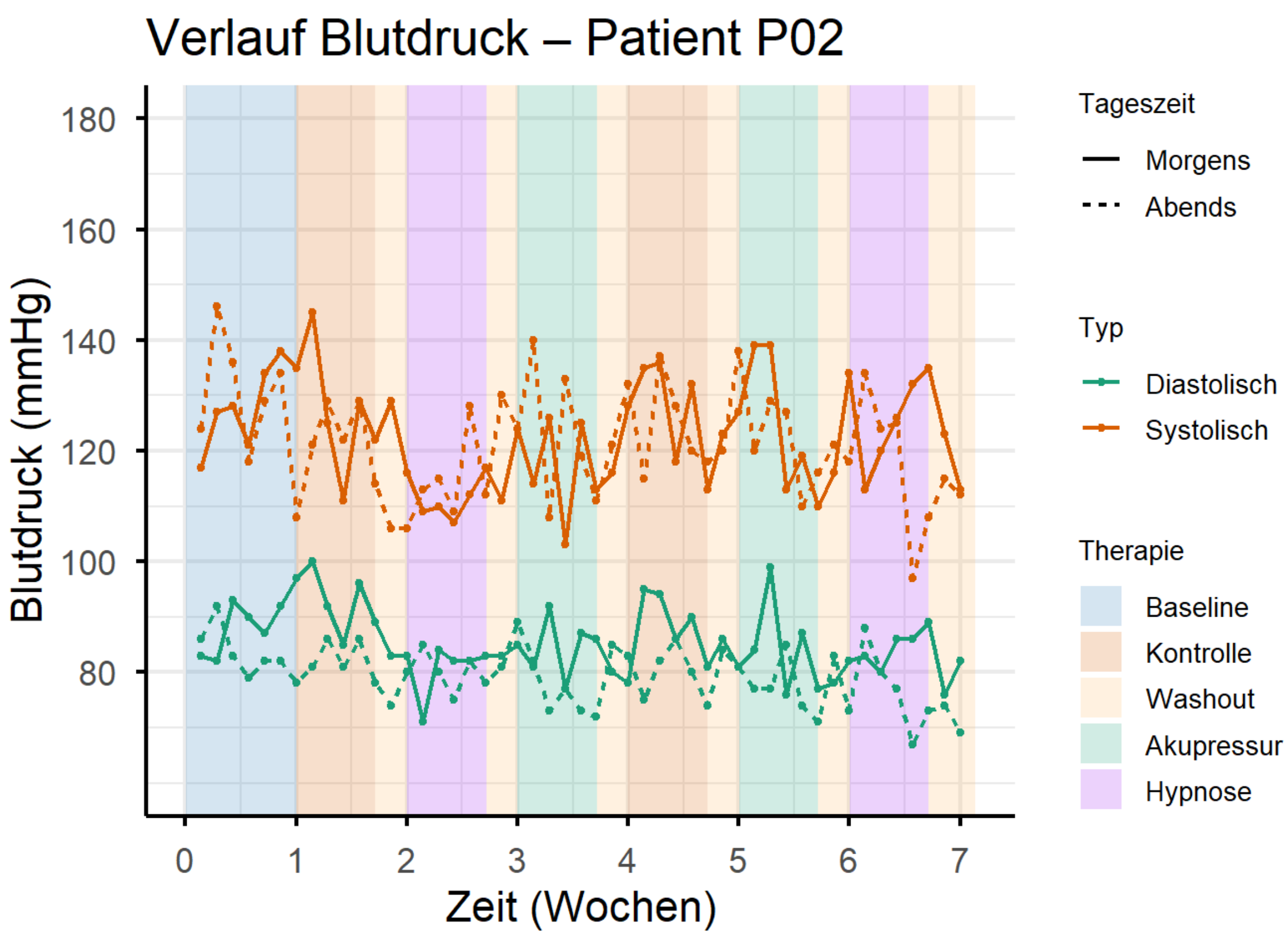

2. Ihre persönlichen Ergebnisse für die Pulsfrequenz

In der Abbildung 2 sehen Sie Ihre persönlichen Pulsfrequenzwerte im Verlauf der gesamten Studie. Rechts neben der Abbildung finden Sie die Erklärung der farblichen Markierungen und Linien.

Abbildung 2: Ihre Pulsfrequenzwerte im Verlauf

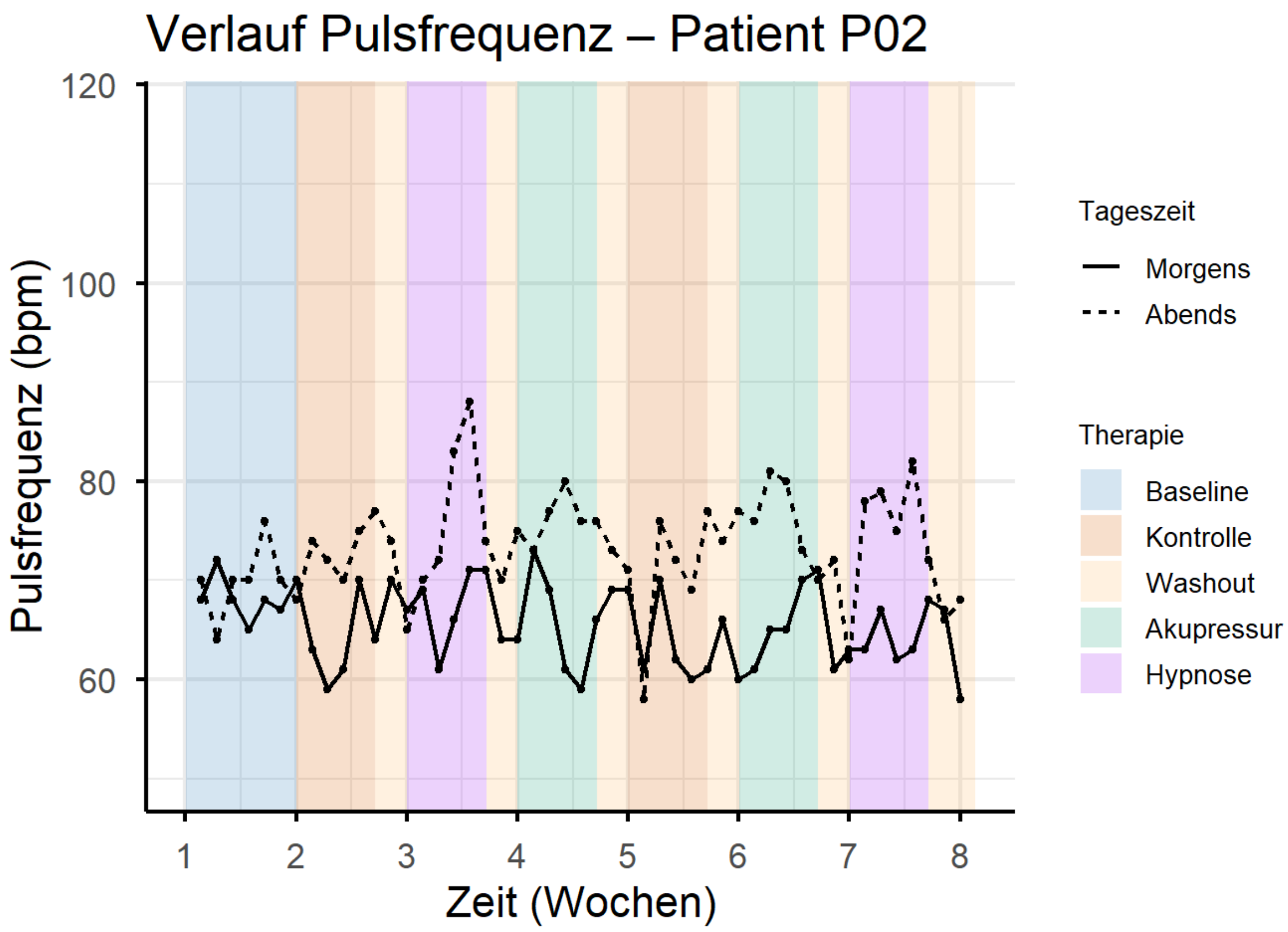

3. Ihre persönlichen Ergebnisse für Stressbelastung, Vitalität und Wohlbefinden

Auf dieser Seite finden Sie die Abbildungen 2 bis 4, die den Verlauf für die tägliche Stressbelastung, Vitalität und Wohlbefinden wiedergeben. Die einzelnen Messpunkte sind mit schwarzen Linien verbunden.

Abbildung 3: Stressbelastung im Verlauf

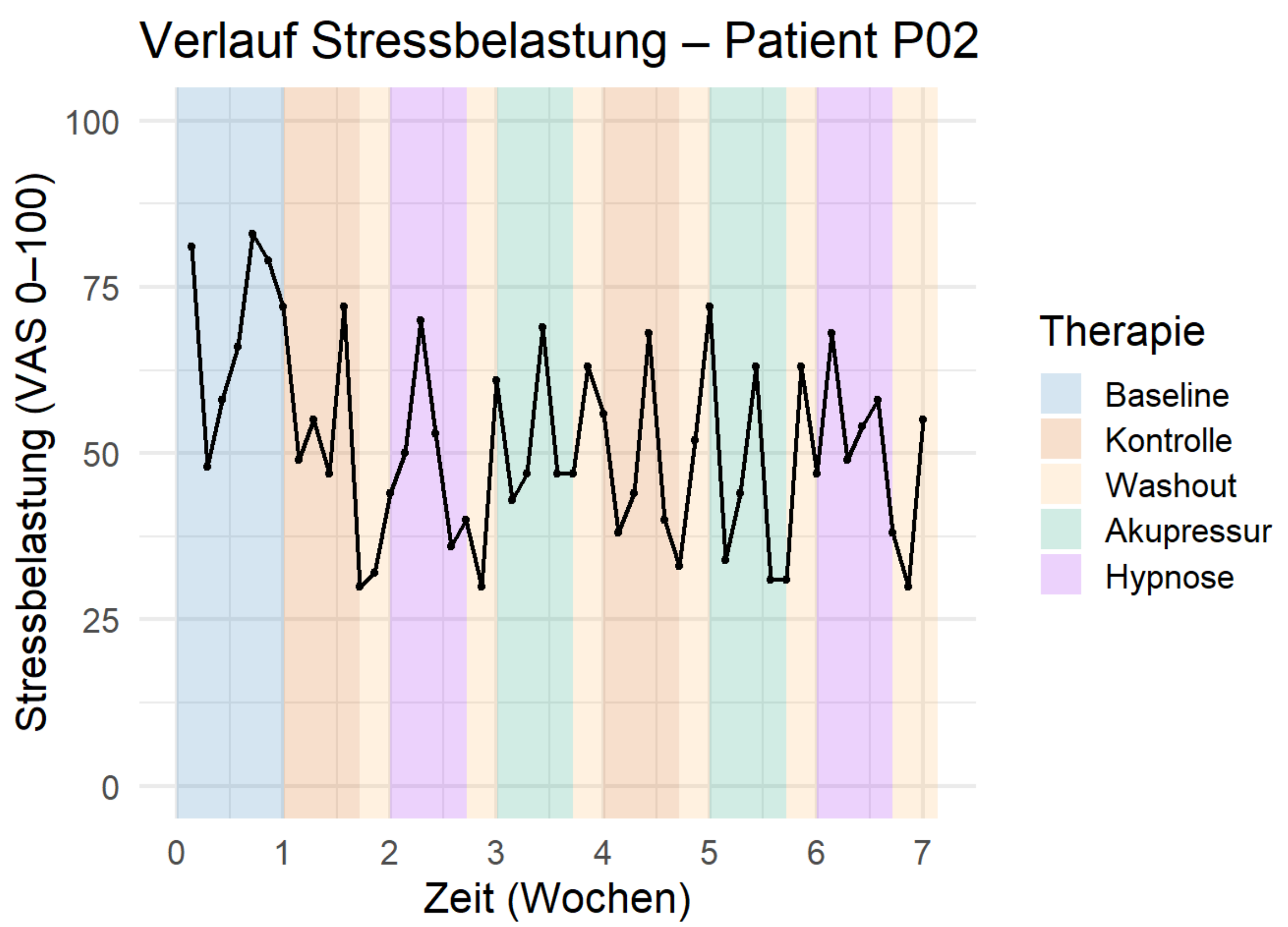

Abbildung 3: Vitalität im Verlauf

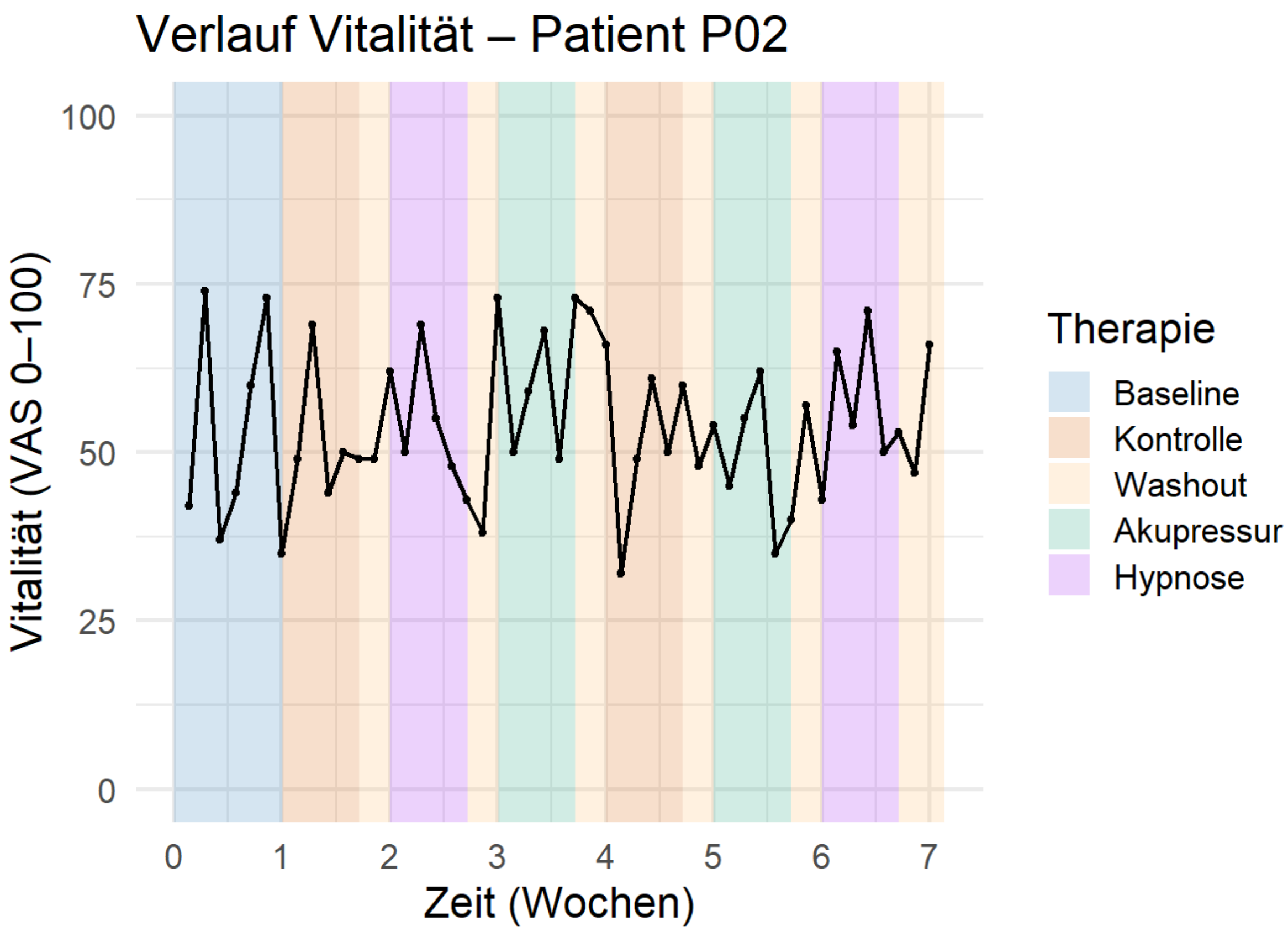

Abbildung 4: Wohlbefinden im Verlauf

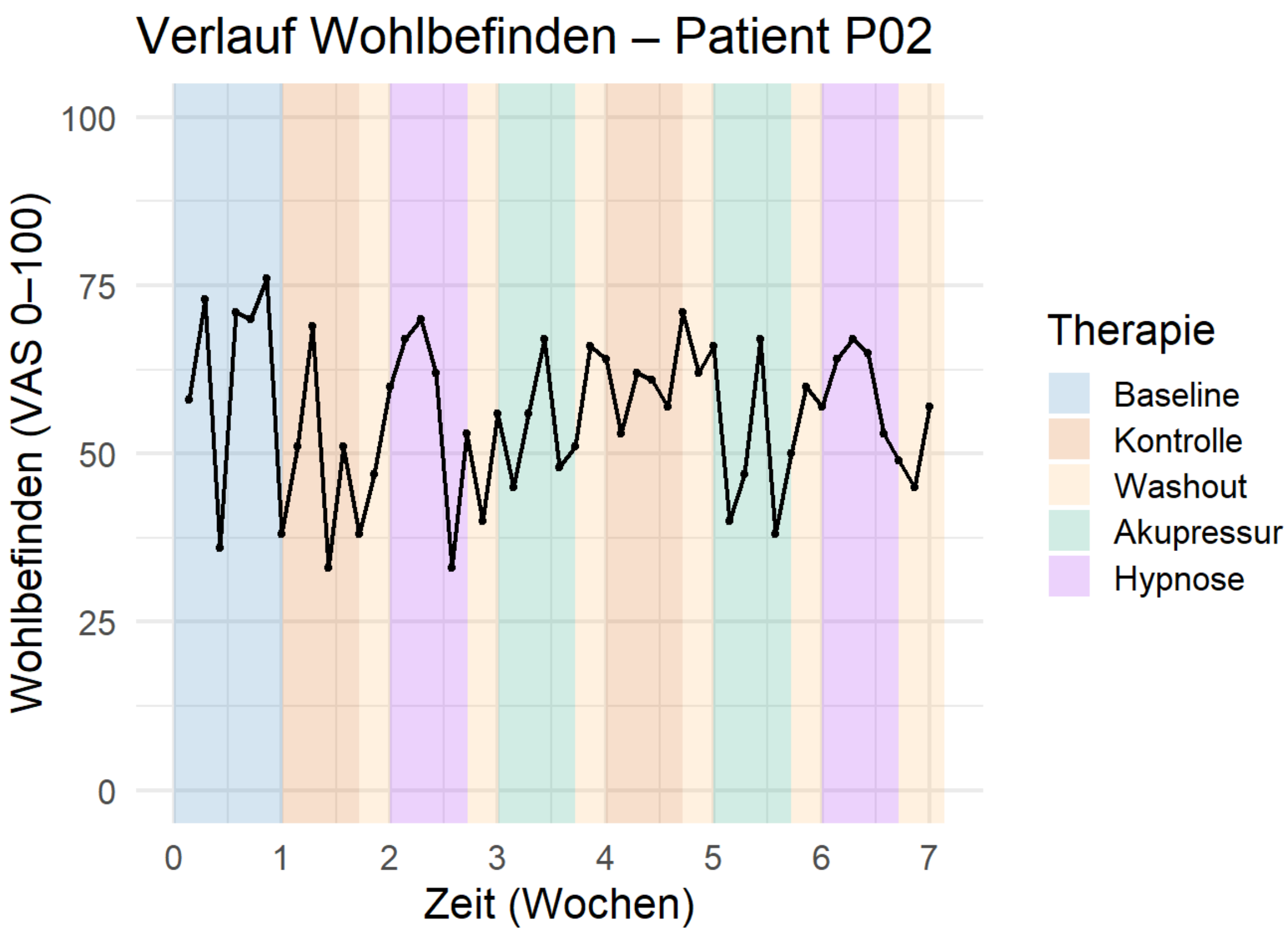

3. Ergebnisse – Mittelwerte über alle Studienteilnehmer

In Abbildung 5 und 6 finden Sie die zusammengefassten Mittelwerte aller Teilnehmer und aller Zeitpunkte der Studie für Blutdruck, Stressbelastung, Vitalität und Wohlbefinden. Die Bezeichnung „Block" meint 1x eine zufallsverteilte Reihenfolge von Akupressur-, Hypnose- bzw. Kontrollphase. Über „Total" sehen Sie die Durchschnittswerte für Zyklus 1 und 2 zusammengefasst. Die Punkte zeigen Werte, die Antennen nach oben und unten zeigen die Varianz der Werte als Standartabweichung (SD).

Abbildung 5: Blutdruck und Puls aller Teilnehmer

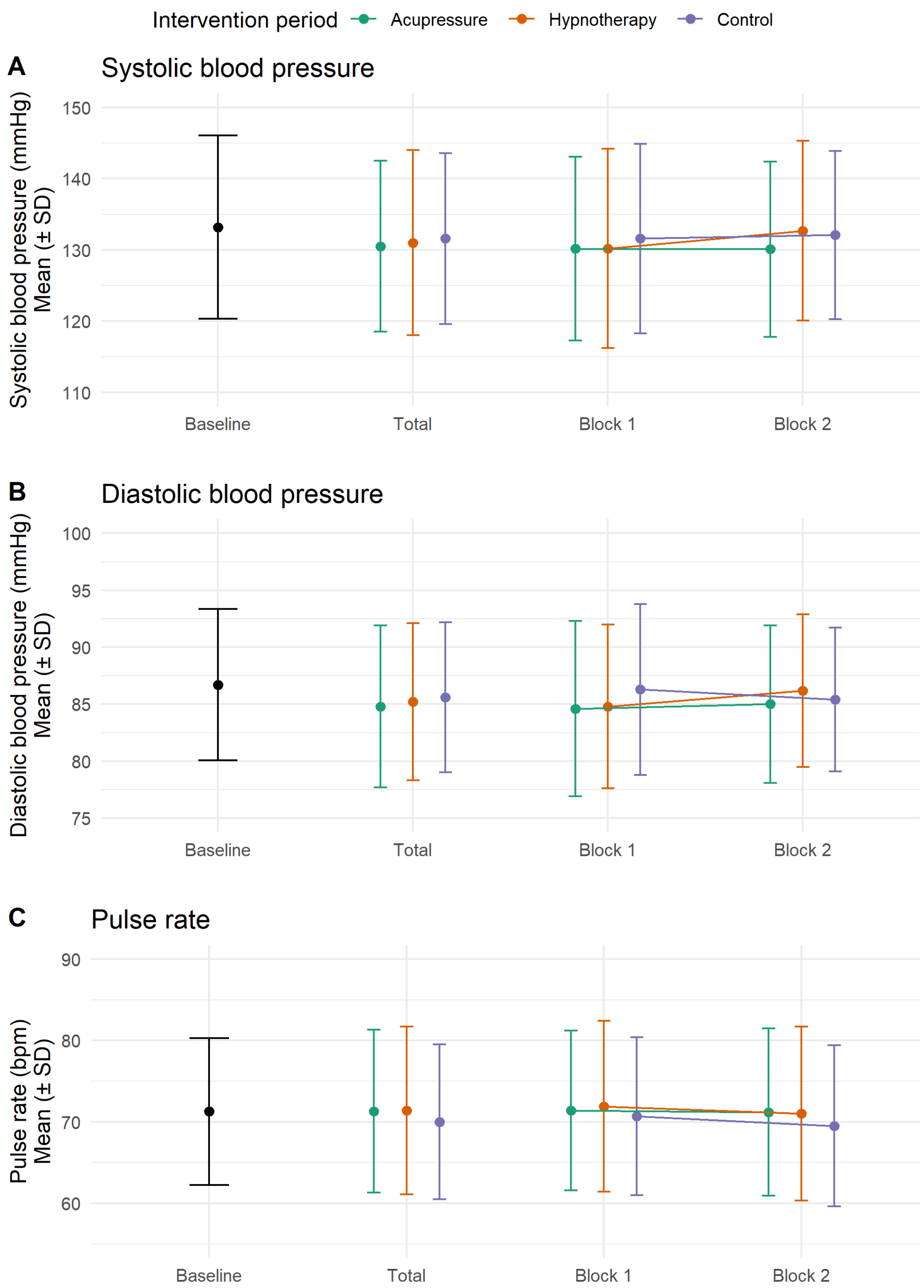

Abbildung 6: Stressbelastung, Vitalität und Wohlbefinden aller Teilnehmer

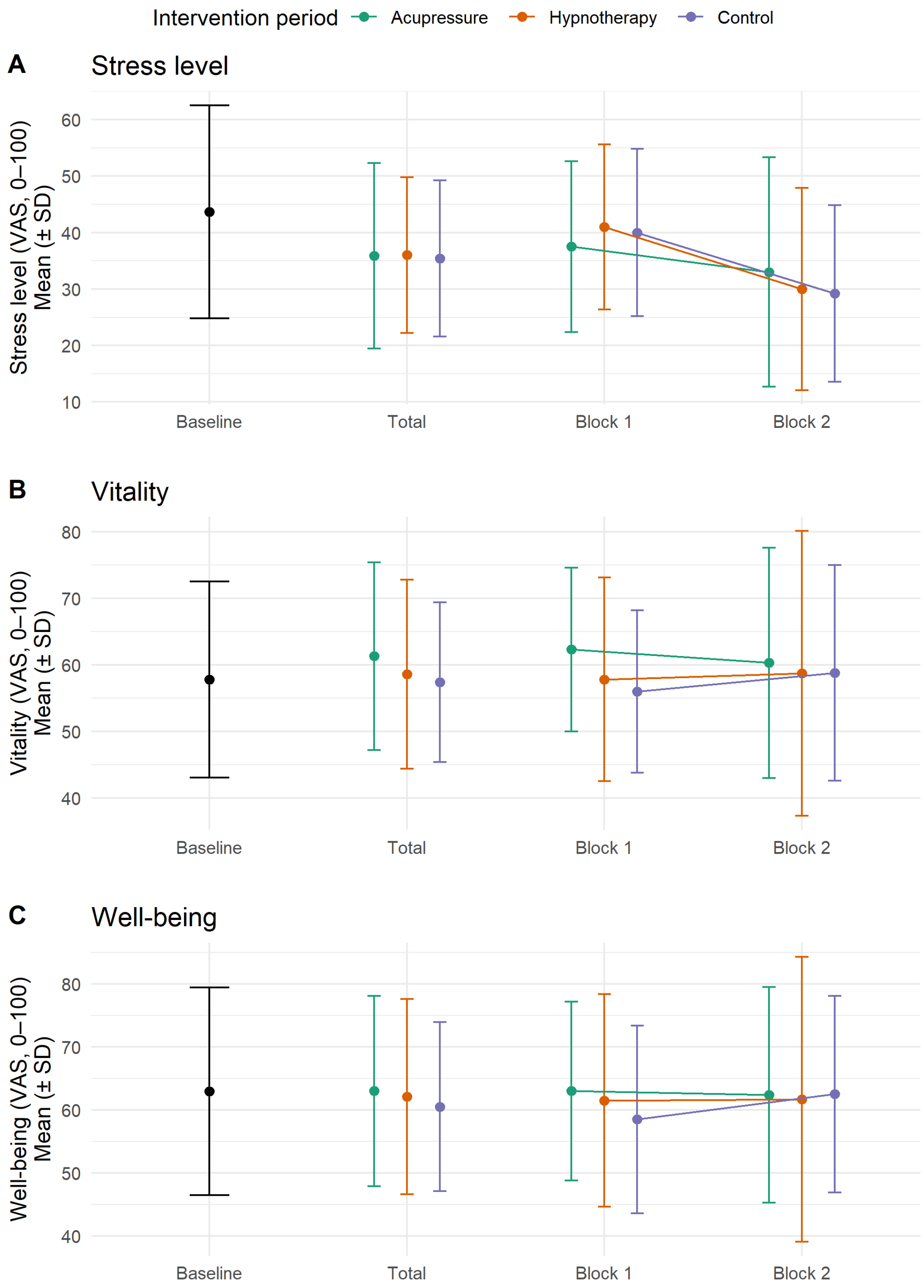

Supplement: Supplementary file 1 [file Supplementary_file_1.pdf]
